# Rotational Scanning Electron Micrographs (rSEM): A novel and accessible tool to visualize and communicate complex morphology

**DOI:** 10.3897/zookeys.328.5768

**Published:** 2013-09-03

**Authors:** David K-B Cheung, Adam J. Brunke, Nesrine Akkari, Carina Mara Souza, Thomas Pape

**Affiliations:** 1 Natural History Museum of Denmark, Zoological Museum, Universitetsparken 15, Copenhagen, Denmark, 2100; 2 Department of Animal Biology, Institute of Biology, State University of Campinas (UNICAMP), Barão Geraldo, Campinas, São Paulo, Brazil, P.O.B. 6109, 13083-970

**Keywords:** Scanning electron microscopy, digital taxonomy, interactive animation

## Abstract

An accessible workflow is presented to create interactive, rotational scanning electron micrographs (rSEM). These information-rich animations facilitate the study and communication of complex morphological structures exemplified here by male arthropod genitalia. Methods are outlined for the publication of rSEMs on the web or in journal articles as SWF files. Image components of rSEMs were archived in MorphBank to ensure future data access. rSEM represents a promising new addition to the toolkit of a new generation of digital taxonomy.

## Introduction

In the effort to discover, describe and organize the planet’s biodiversity, taxonomists are faced with the challenge of providing clear and concise diagnostic characters in species descriptions, often involving structures with complex morphology. To address this challenge, a variety of imaging methods have emerged over the past ten years to complement more traditional line drawings, which continue to play an important role in species descriptions. Traditionally, diagnostic characters are illustrated in one or a set of standard views to facilitate the comparison of taxa within and between publications by taxonomists and other users. In this current paradigm, characters not or suboptimally visible in these conventional views are difficult for taxonomists to convey and for users to interpret, placing a constraint on the range of published morphological data. These issues are further exacerbated in the case of complex or asymmetrical male genitalia, which are challenging to clearly and accurately describe in words. Multifocal, ‘stacked’ images of external structures or habitus are now standard in most taxonomic descriptions and recent advances in micro-computed tomography (µCT) and magnetic resonance imaging (MRI) promise to yield exciting new character data from both external and internal morphology (i.e., Hart et al. 2003, Beutel et al. 2008, Mietchen et al. 2008); even from non-sclerotized specimens preserved in alcohol (Faulwetter et al. 2013) or living specimens (Lowe et al. 2013). Both µCT and MRI scans can be represented as rotating (Mietchen et al. 2008), even interactive (Faulwetter et al. 2013) animations and have dramatically increased the level of morphological data available to users in one ‘illustration’. Raman-atomic force microscopy has revealed morphological differences in molecular surface structure between various Diptera taxa, at the nanometer level of resolution (Andersen and Gaimari 2003).

Since the early 1970s (for an early example, see Herman 1972), scanning electron microscopy (SEM) has had an increasingly important role in several taxonomic fields, especially the study of Arthropoda, in exploring the fine surface sculpture and other morphological aspects impossible to assess with a standard stereo- or compound microscope, or poorly resolved at higher magnifications using µCT and MRI (e.g., Rota 2005; Whitmore 2009, 2011; Akkari and Enghoff 2011, 2012; Grzywacz et al. 2012; Miller et al. 2012; Shear 2012). For some millipede families (i.e., Dalodesmidae Cook, 1896, Haplodesmidae Cook, 1896) characterized by minute genitalia, descriptions of new taxa are based primarily on SEM, supplemented with line drawings (e.g., Mesibov 2008, 2009). Additionally, the level of detail and magnification provided by SEMs are now commonly harnessed to facilitate the interpretation of phylogenetic character states (e.g. Edgecombe and Hollington 2005, Chani-Posse 2013).

Recently, we have sought for an accessible method to integrate and present diagnostic differences of taxa existing at non-standard and standard angles, using SEM micrographs. Here we describe an SEM image workflow that results in information-rich, rotatable animations (hereafter referred to as rSEMs – rotational scanning electron micrographs), which can be either web published or embedded in PDF articles for publication in journals accepting such media (e.g., ZooKeys). Examples of rSEM from different arthropod groups (Coleoptera, Diptera, Myriapoda) are provided.

## Specimen preparation and mounting

**Figure 1.**
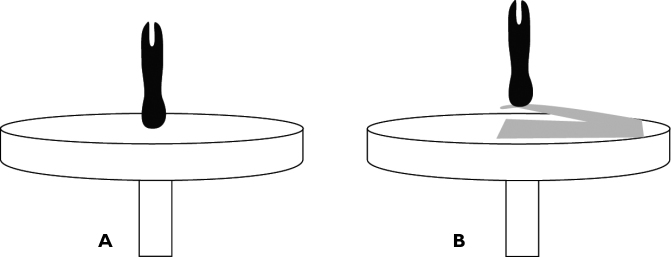
Specimens directly (**A**) and secondarily (**B**) mounted on an aluminum SEM stub.

Larger, dry specimens were mounted (see below) without special cleaning or dehydration. Specimens preserved in 70% ethanol or glycerin were first cleaned of debris and then dehydrated in 96% ethanol, then acetone, and air dried before mounting. Fragile specimens (e.g. small flies, nymphs, larvae) should be dried using critical point drying to avoid shriveling or collapsing (Oster and Pollister 1966). Specimens with a broad, flattened base can be mounted directly onto the SEM stub (Fig. 1A). The majority of specimens will have a much smaller contact point with the stub and should be attached via flexible secondary mounts consisting of electrical tape or thin, aluminum wire (Fig. 1B). Specimens should be mounted as close to the center of rotation and as perpendicular to the SEM stub surface as possible (Fig. 1), to reduce image alignment difficulties and ‘swaying’ rSEMs. Secondary mounts allow manipulation of the specimen to the optimal position, even after coating.

### Image Acquisition and processing

**Figure 2.**
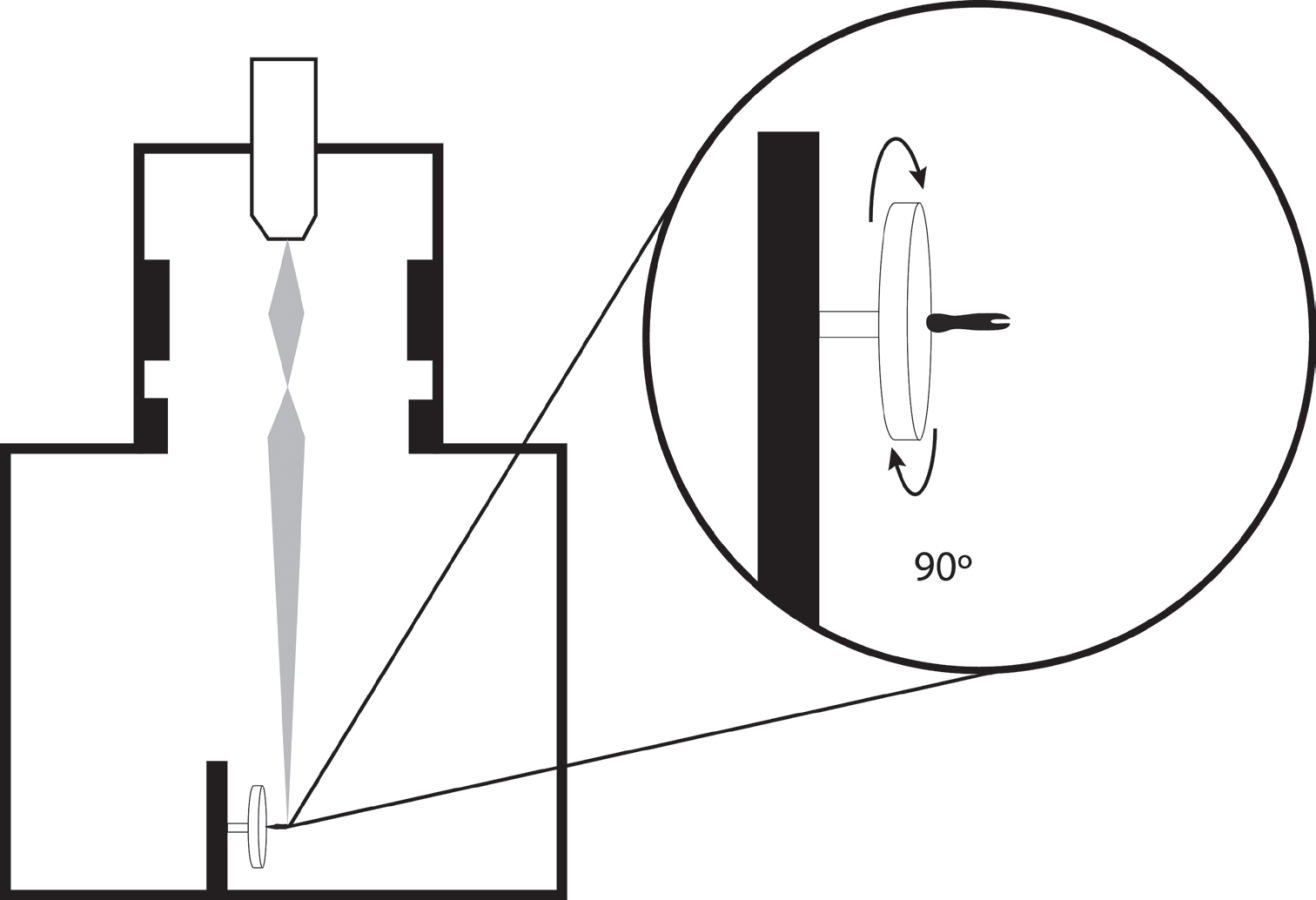
Schematic representation of SEM specimen and stage positioning for creation of rSEM image frames.

For users unfamiliar with the elements of this workflow, a step-by-step set of instructions is provided in provided in Appendix 1. Specimens were sputter-coated with platinum/palladium and studied with a JEOL JSM-6335F scanning electron microscope. Note that the microscope stage MUST be able to tilt to 90° in order to produce a usable animation (Fig. 2). Images were taken at fixed rotational intervals and named sequentially.

Images were then processed (adjusting the exposure, contrast, highlights, shadows, whites, blacks) and cropped to improve image quality and detail, and to ensure that all images were ‘aligned’, such that they form a smooth transition when browsed in sequence. Images were exported as JPG. Image processing can be accomplished using subscription based software such as Adobe® Lightroom® or Photoshop®, or open-source software such as ImageJ (http://rsbweb.nih.gov/ij/). The images can now be integrated into an animation for submission to a scientific journal or published on a website. The individual images generated for this study were archived in MorphBank under object #830913, 830920-830935, 830953-830970 and 831016-831034; they can be found together by searching for collection ‘rSEM’.

### Creation of rSEMs for submission to a journal

rSEMs in the present article and in Akkari et al. (2013) were submitted as SWF files. These files were embedded in the PDF version of each article by the publisher and therefore instructions for this procedure are not included here. Depending on the journal, SWF files may be embedded or included as downloadable, supplementary figures (Appendix 1 and 2). We have opted for the integrated nature of embedded files as plates rather than disjointed supplementary files. The creation of rSEMs was performed using Adobe® Flash® CS5, which were then exported as SWF files. Images were inserted as individual key frames and animated using the following Action Script in the action palette (script by ‘winlwin’, http://www.icodesnip.com/snippet/actionscript-3/360-degree-view):

import flash.events.Event;

**instancename**.stop();

var frameTo:Number=0;

addEventListener(Event.ENTER_FRAME,goTo);

function goTo(e:Event):void {

    frameTo=int(mouseX/stage.stageWidth*instancename.totalFrames)+1;

    **instancename**.gotoAndStop(frameTo);

}

### Web publication of rSEM

**Figure 3.**
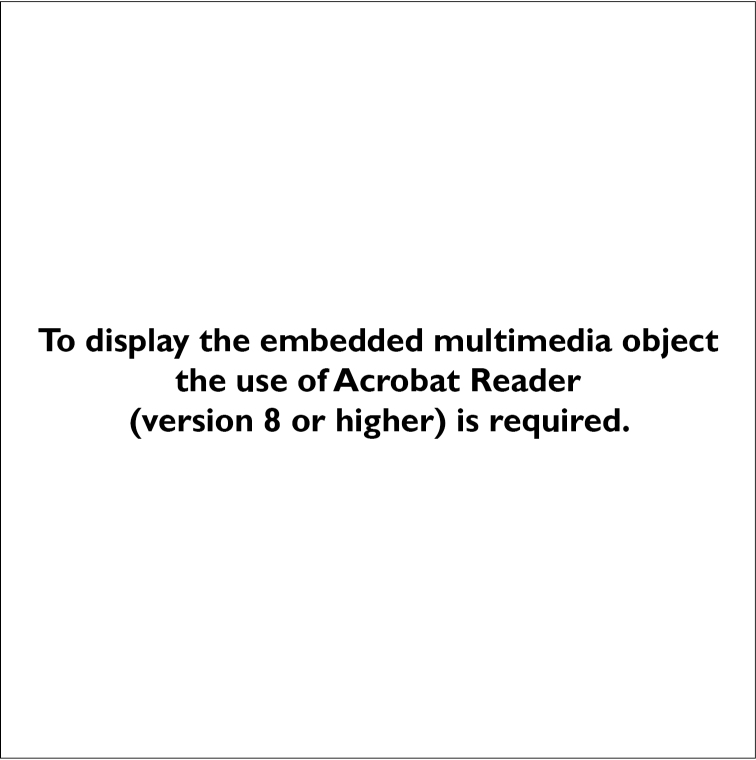
rSEM illustrating the distiphallus of *Oxysarcodexia (Xylocamptopsis) fringidea* (Curran & Walley) (Sarcophagidae, Diptera). The offline version of this rSEM can be explored by moving the mouse over the image in the x-axis and can be viewed using Adobe® Acrobat® Reader version 8 or higher, with Adobe® Flash® Player installed. Compatible with Windows and Mac OS X.6 (Snow Leopard, 2009) or newer. Image components were archived in MorphBank as objects #830953-830970.

**Figure 4.**
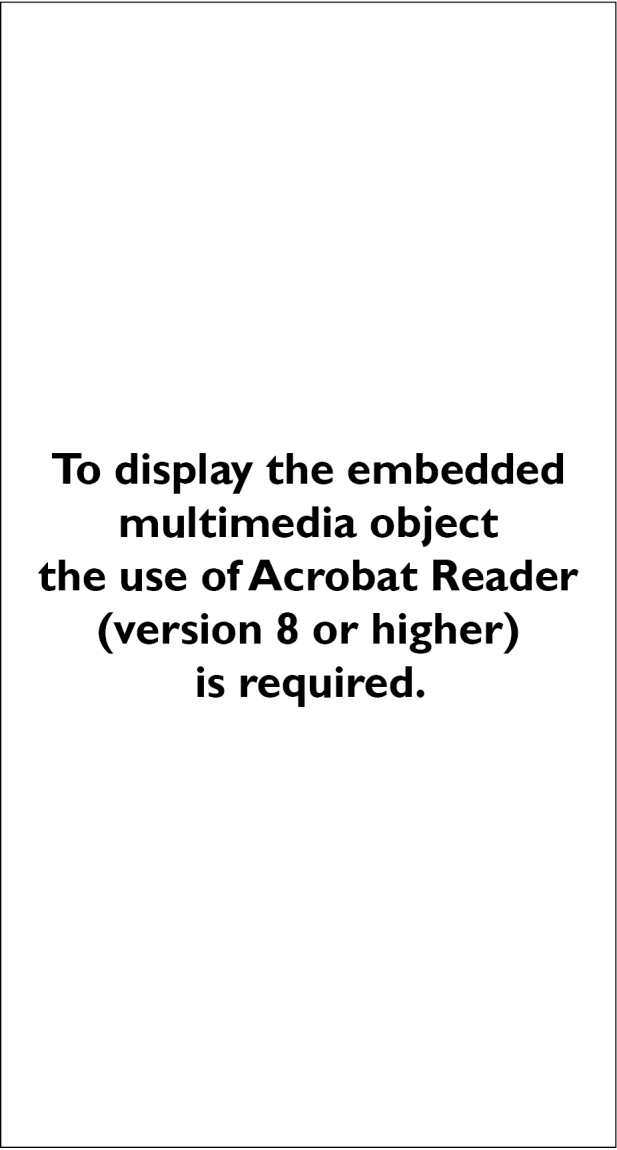
rSEM illustrating the posterior gonopod of *Ommatoiulus khroumiriensis* Akkari & Enghoff (Julidae, Diplopoda). The offline version of this rSEM can be explored by moving the mouse over the image in the x-axis and can be viewed using Adobe® Acrobat® Reader version 8 or higher, with Adobe® Flash® Player installed. Compatible with Windows and Mac OS X.6 (Snow Leopard, 2009) or newer. Image components were archived in MorphBank as objects #831016-831034.

**Figure 5.**
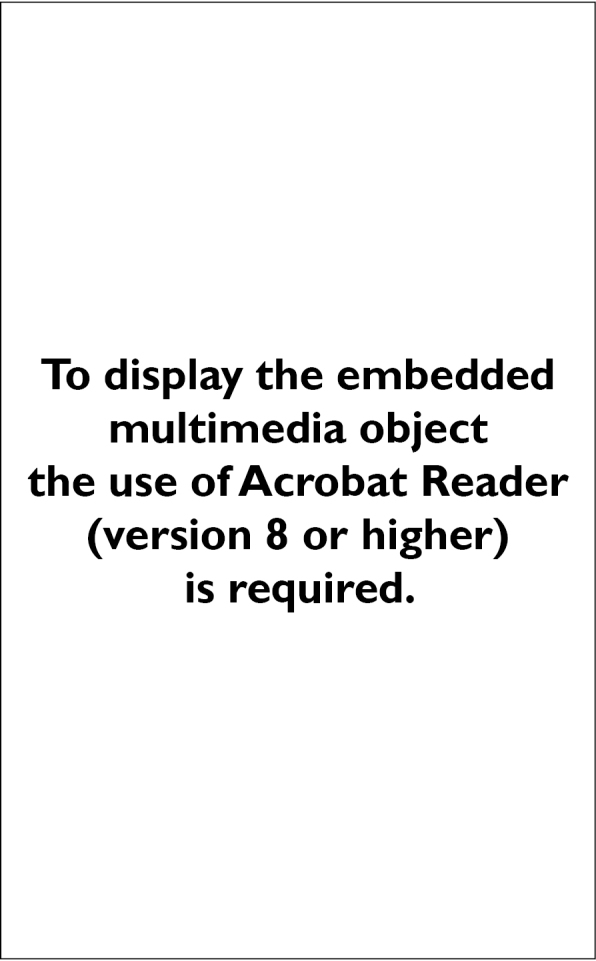
rSEM illustrating the median lobe (paramere removed) of *Bolitogyrus* sp. (Staphylinidae, Coleoptera). The offline version of this rSEM can be explored by moving the mouse over the image in the x-axis and can be viewed using Adobe® Acrobat® Reader version 8 or higher, with Adobe® Flash® Player installed. Compatible with Windows and Mac OS X.6 (Snow Leopard, 2009) or newer. Image components were archived in MorphBank as objects #830913, and 830920-830935.

To publish an rSEM on the web, a program or web plug-in is needed to integrate the images into an animation. There is a multitude of these available online of varying quality and functionality (search ‘object viewer’ or ‘360 panorama’) ranging from subscription-based (Magic 360™ (http://www.magictoolbox.com/magic360/)) to free and open-source (Reel© 1.2.1 (http://jquery.vostrel.cz/reel#dict)). A set of scripts needs to be downloaded and a folder containing the sequentially numbered images should be created. We have tested the two previously mentioned options and both result in web animations that are compatible with all operating systems, including mobile devices. For full instructions, readers are directed to the tutorials provided by the publishers (see links above). The rSEMs included in the html version of Akkari et al. (2013) and the present paper (Figs 3–5) were created using Reel 1.2.1, and an example of Magic 360 output is given in Appendix 2.

## Discussion

The workflow described herein will generate interactive, information-rich animations that facilitate both the study and communication of complex morphology in systematics (Figs 3–5). rSEMs combine sharply detailed SEM micrographs into a single, holistic representation of a structure that may even reveal diagnostic characters visible but yet unnoticed when using a light microscope. The use of SEM is not always optimal or appropriate as users may not have access to facilities or cannot image large, fragile or unique specimens. In these cases, morphological characters will still need to be illustrated by line drawings or stacked images, despite their potential complexity. Differences in color and degree of sclerotization are also lost in SEM. However, rSEMs can be created using technology and software in widespread use and provide an accessible alternative to other imaging methods such as µCT if the user is focused on structural aspects of external morphology. As HTML5 is becoming more prominent than Flash® as a media development standard, an update is planned to incorporate instructions for developing rSEM in HTML5. rSEMs are integrated into a taxonomic revision for the first time in a companion article (Akkari et al. 2013) to provide clear, accurate and user-accessible descriptions of morphological structures, which are typically difficult to interpret without taxonomic expertise.

With the development of Web 2.0 and Open Access publishing, the demand for image quality and methods for visualizing taxonomic traits has increased. Recently, publishing 3D models and embedding multimedia files in biomedical journals has become common practice (e.g. Tyzack 2008, Ziegler et al. 2011). However, the adoption of such media in taxonomy is still relatively scattered, and is mostly limited to MRI and Micro-computed Tomography (e.g. Mietchen et al. 2008, Boistel et al. 2011, Laforsch et al. 2012, Faulwetter et al. 2013). This may be due to the perception that sophisticated imaging requires special software, e-infrastructure and significant funding. The rSEM workflow provided here demonstrates this to be incorrect. The workflow does not require special equipment beyond that of a scanning electron microscope and the processing and integration of images can be accomplished using photo editing software and Adobe Flash CS5 or a plug-in such as Reel 1.2.1.

Here, additional rSEMs of arthropod genitalia were provided as examples of complex morphology. However, this approach could easily be extended to structures than other genitalia, and could be applied to the illustration of a phylogenetically important character system (e.g., a rotatable insect head capsule). So far, we have only animated rSEM in the x-axis because our test structures varied mainly along the y-axis. However, illustration of structures along both x and y-axes of rotation, while requiring many more images, may be a powerful form of data delivery in certain situations. For example, if a finely controlled, electronic stage is available, full-color, rotatable habitus images could be produced using a dissecting microscope and stacking software. There are clearly far more applications of rSEM or rSEM-like animations than we have presented here, and we encourage readers to experiment with this technique in their own scientific exploration of Earth’s biodiversity.

## Appendix 1

New user guide to the creation and publication of rSEM (doi: 10.3897/zookeys.328.5768.app1) File format: Microsoft Word document (doc).

**Explanation note:** A step-by-step style of instructions for new users, involving the software used by the authors. Note: in the future, these instructions may not correspond exactly with the interfaces of the corresponding software.

## Appendix 2

rSEM illustrating the distiphallus of *Oxysarcodexia (Xylocamptopsis) fringidea* (Curran & Walley) (Sarcophagidae, Diptera); web-published using Magic 360^TM^ script files. Click and drag to rotate the rSEM and point click to open and close the magnification tool. (doi: 10.3897/zookeys.328.5768.app2) File format: Hypertext Markup Document, archived (zip).

**Explanation note:** Example of rSEM published online using Magic 360^TM^ script.
