# Supplementary material for: Rotational Scanning Electron Micrographs (rSEM): A novel and accessible tool to visualize and communicate complex morphology
Source: Zookeys. 2013 Sep 3;(328):47–57. doi: 10.3897/zookeys.328.5768 (PMC3800821; doi:10.3897/zookeys.328.5768)
Supplement: Supplementary file 1 — New user guide to the creation and publication of rSEM (doi: 10.3897/zookeys.328.5768.app1) File format: Microsoft Word document (doc). [file ZooKeys-328-047-s001.doc]

**Appendix I**

**New user guide to the creation and publication of rSEM**

Note: the following instructions were developed for use with the specific programs used by the authors. For other software options, please see the main body of the text.

**Specimen preparation and mounting**

Larger, dry specimens were mounted (see below) without special cleaning or dehydration. Specimens preserved in 70% ethanol or glycerin were first cleaned of debris and then dehydrated in 96% ethanol, acetone, air dried before mounting. Fragile specimens (e.g. small flies, nymphs, larvae) should be dried using critical point drying to avoid shriveling or collapsing (Oster and Pollister, 1966). Specimens with a broad, flattened base can be mounted directly onto the SEM stub (Fig. 1A). The majority of specimens will have a much smaller contact point with the stub and should be attached via flexible secondary mounts consisting of electrical tape or thin, aluminum wire (Fig. 1B). Specimens should be mounted as close to the center of rotation and as perpendicular to the SEM stub surface as possible (Fig. 1), to reduce image alignment difficulties and ‘swaying’ rSEMs. Secondary mounts allow manipulation of the specimen to the optimal position, even after coating.

**Image Acquisition**

The following steps were followed to produce images for the rSEM:

1. Tilt the stage (and specimen) to 90˚ and set contrast, brightness, and magnification (Fig. 2).

2. Rotate the specimen to a perfect dorsal, ventral or lateral view and focus the image.

3. Create this first image and remember to name the images sequentially, starting with ‘01’.

4. Chose the number of images to capture, depending on the desired level of smoothness for your rSEM (we have found that 18 images generally provide a balance between detail and time investment.)

5. Rotate the specimen toward the left (clockwise in the SEM chamber) by the appropriate number of degrees (see Table S1), refocus and adjust brightness/contrast if they have shifted in this new position. Capture your image. **NOTE:** rotating the specimen toward the right will result in reversed animation controls in the final rSEM.

6. Repeat step five until all frames are acquired.

**Table 1.** Stage rotation intervals for the creation of rSEM animations based on the number of image frames desired.

| Number of images | 9 | 18 | 36 | 72 |
| --- | --- | --- | --- | --- |
| Rotation interval | 40° | 20° | 10° | 5° |

**Image processing**

Images were then processed in Adobe Lightroom 4 (Adobe Systems Inc., San Jose) by adjusting the tone and tone curve options (exposure, contrast, highlights, shadows, whites, blacks) for the first image to improve image quality and detail. The Crop tool was used to exclude unwanted background. These settings were then applied to the entire image set using the following steps (Fig. S1):

1. Switch to grid view.

2. Select the first frame and then group select the entire set of images.

3. Click the ‘Sync settings’ button in the lower right corner.

4. In the settings menu, check the Tone Curve box, Aspect Ratio box and all boxes under Basic Tone.

5. Select ‘Synchronize’.

6. Review each image for minor adjustments to tone settings or rotation of the subject using the Crop tool, to achieve a smooth transition when browsing between images in sequence.


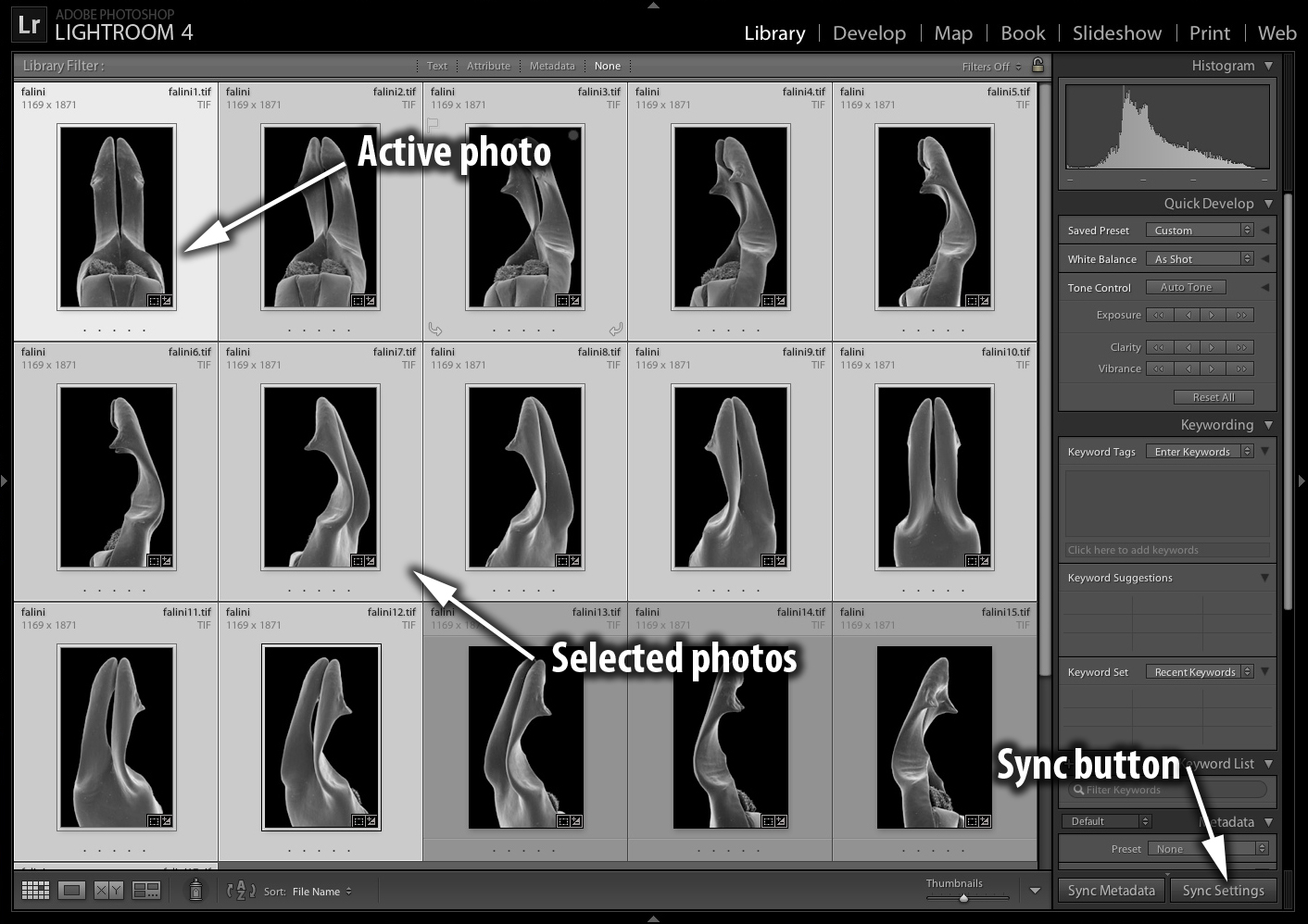


Figure S1. Using the ‘Sync settings’ function in Adobe® Lightroom® 4.

*Optional technique for achieving uniform and deep black backgrounds*

1. Using the brush tool in the Development module, chose the following settings (see Fig. S2): exposure = −4; shadows = −100, auto mask = checked; feather = 30; shadow clipping = activated.

2. Paint over the background using the brush tool.

3. Deactivate shadow clipping, and repeat steps 1 and 2 for each image.

To export, select all images and export in JPG format. The images can now be integrated into an animation for submission to a scientific journal or published on a website.

**
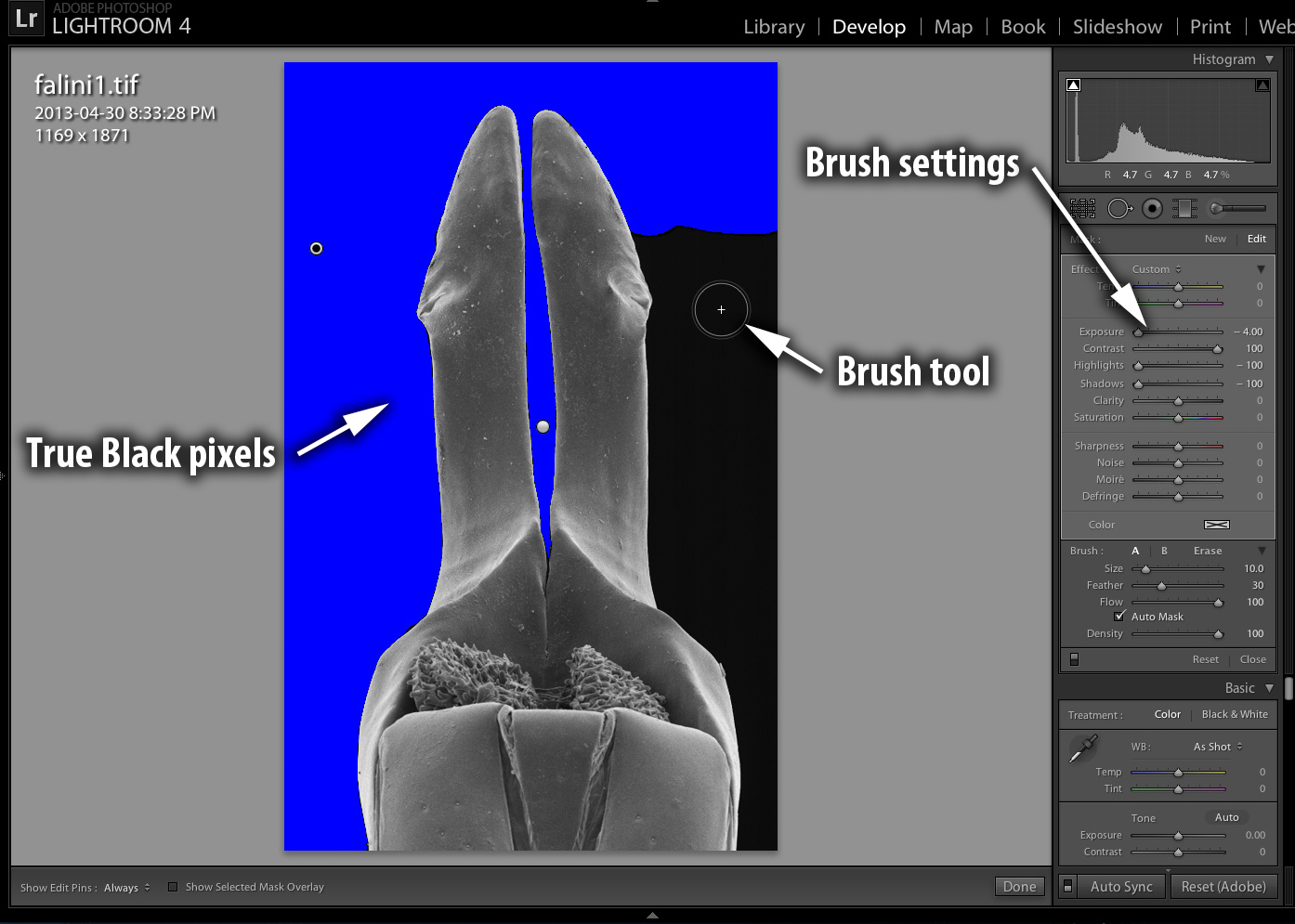
**

Figure S2. Painting a background mask to achieve uniform, true black backgrounds in Adobe® Lightroom® 4.

**Creation of rSEMs for submission to a journal.**

rSEMs in the present article and in Akkari et al. (2013) were submitted as SWF files. The creation of rSEMs as SWF files was performed using the following procedure in Adobe® Flash® CS5:

1. Create a new Actionscript 3.0 document with the exact dimensions as an image in your image set.

2. Go to Insert -> New Symbol.

3. Under Type, select Movie Clip, click OK.

4. In the timeline (Fig. S3), right click on frame 2 and select Insert Blank Key Frame.

5. Insert a blank key frame for every image in your set.

6. Left click on frame 1 in the timeline to make it active.

7. Click on File-> Import to Library. Select your set of images and open them.

8. Open the Library palette by clicking on Window-> Library.

9. Click and drag your first image from the Library palette onto the stage.

10. Center the image by selecting it; go to the Properties palette.

10. Under Position and Size, set the x and y values to 0.

11. Go to the second frame by hitting the “>” key on the keyboard.

12. Repeat steps 9–11 until all the images in your set are in their own keyframe.

13. Click on the Scene button under the timeline (Fig. S3) to return to Scene 1.

14. The stage should be empty at this point.

15. In the Library palette, click and drag Symbol 1 onto the stage and center it using the Properties palette.

16. With the New Symbol still selected on the stage, go to Properties palette and type in ‘imgrotate’ instead of ‘<Instance Name>’.

17. Go to Window-> Actions to open the Actions palette.

18. Go to Insert-> Timeline-> Layer.

19. Left click on frame 1 of layer 2. Inside the Action palette, paste in the following code:

import flash.events.Event;

imgrotate.stop();

var frameTo:Number=0;

addEventListener(Event.ENTER_FRAME,goTo);

function goTo(e:Event):void{

frameTo=int(mouseX/stage.stageWidth*imgrotate.totalFrames)+1;

imgrotate.gotoAndStop(frameTo);

}

20. Test your movie by going to Control -> Test Movie -> Test.

21. Your rSEM should now rotate with the movement of your mouse on the x-axis.

22. Save and export your SWF file by going to File-> Export Movie. Select SWF Movie as the format.

**
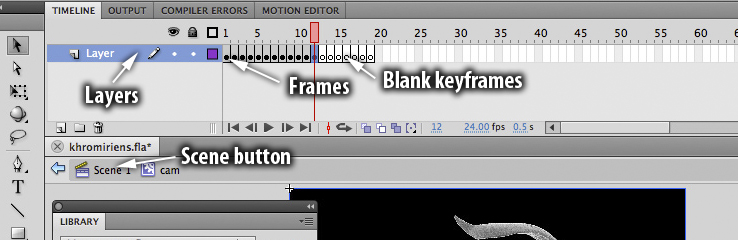
**

Figure S3. Elements of the timeline and the scene selection button in Adobe® Flash ® CS5.

**Web publication of rSEM**

To publish an rSEM on the web, a program or web plug-in is needed to integrate the images into an animation. There is a multitude of these available online of varying quality and functionality (search ‘object viewer’ or ‘360 panorama’) ranging from subscription-based (Magic 360™ (http://www.magictoolbox.com/magic360/)) to free and open-source (Reel© 1.2.1 (http://jquery.vostrel.cz/reel#dict)). A set of scripts needs to be downloaded and a folder containing the sequentially numbered images should be created. We have tested the two previously mentioned options and both result in web animations that are compatible with all operating systems, including mobile devices. For full instructions, readers are directed to the tutorials provided by the publishers (see links above). The rSEMs included in the online version of Akkari et al. (2013) and the present paper (Fig. 3-5) were created using Reel 1.2.1, and an example of Magic 360 output is given in Appendix II.
