# Supplementary material for: Rotational Scanning Electron Micrographs (rSEM): A novel and accessible tool to visualize and communicate complex morphology
Source: Zookeys. 2013 Sep 3;(328):47–57. doi: 10.3897/zookeys.328.5768 (PMC3800821; doi:10.3897/zookeys.328.5768)
Supplement: Supplementary file 2 — rSEM illustrating the distiphallus of Oxysarcodexia (Xylocamptopsis) fringidea (Curran & Walley) (Sarcophagidae, Diptera); web-published using Magic 360TM script files. Click and drag to rotate the rSEM and point click to open and close the magnification tool. (doi: 10.3897/zookeys.328.5768.app2) File format: Hypertext Markup Document, archived (zip). [file ZooKeys-328-047-s002.zip › Fig S4 - magic360/magic360example.html]

Untitled Document
