# Supplementary figures and images for: Rotational Scanning Electron Micrographs (rSEM): A novel and accessible tool to visualize and communicate complex morphology
Source: Zookeys. 2013 Sep 3;(328):47–57. doi: 10.3897/zookeys.328.5768 (PMC3800821; doi:10.3897/zookeys.328.5768)

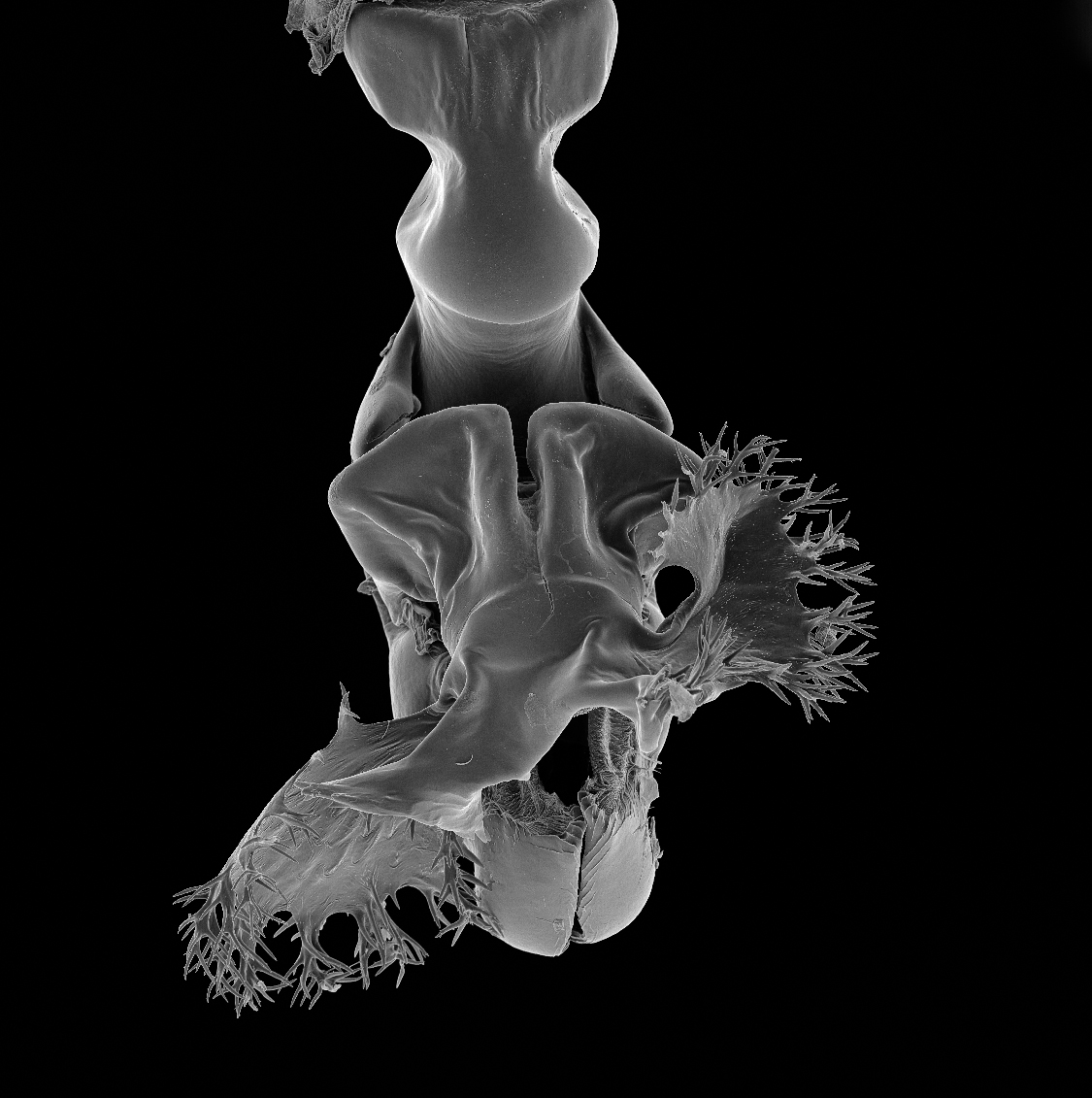

Supplement: Supplementary file 2 — rSEM illustrating the distiphallus of Oxysarcodexia (Xylocamptopsis) fringidea (Curran & Walley) (Sarcophagidae, Diptera); web-published using Magic 360TM script files. Click and drag to rotate the rSEM and point click to open and close the magnification tool. (doi: 10.3897/zookeys.328.5768.app2) File format: Hypertext Markup Document, archived (zip). [file ZooKeys-328-047-s002.zip › Fig S4 - magic360/fringidea/fringidea-lg-01.jpg]

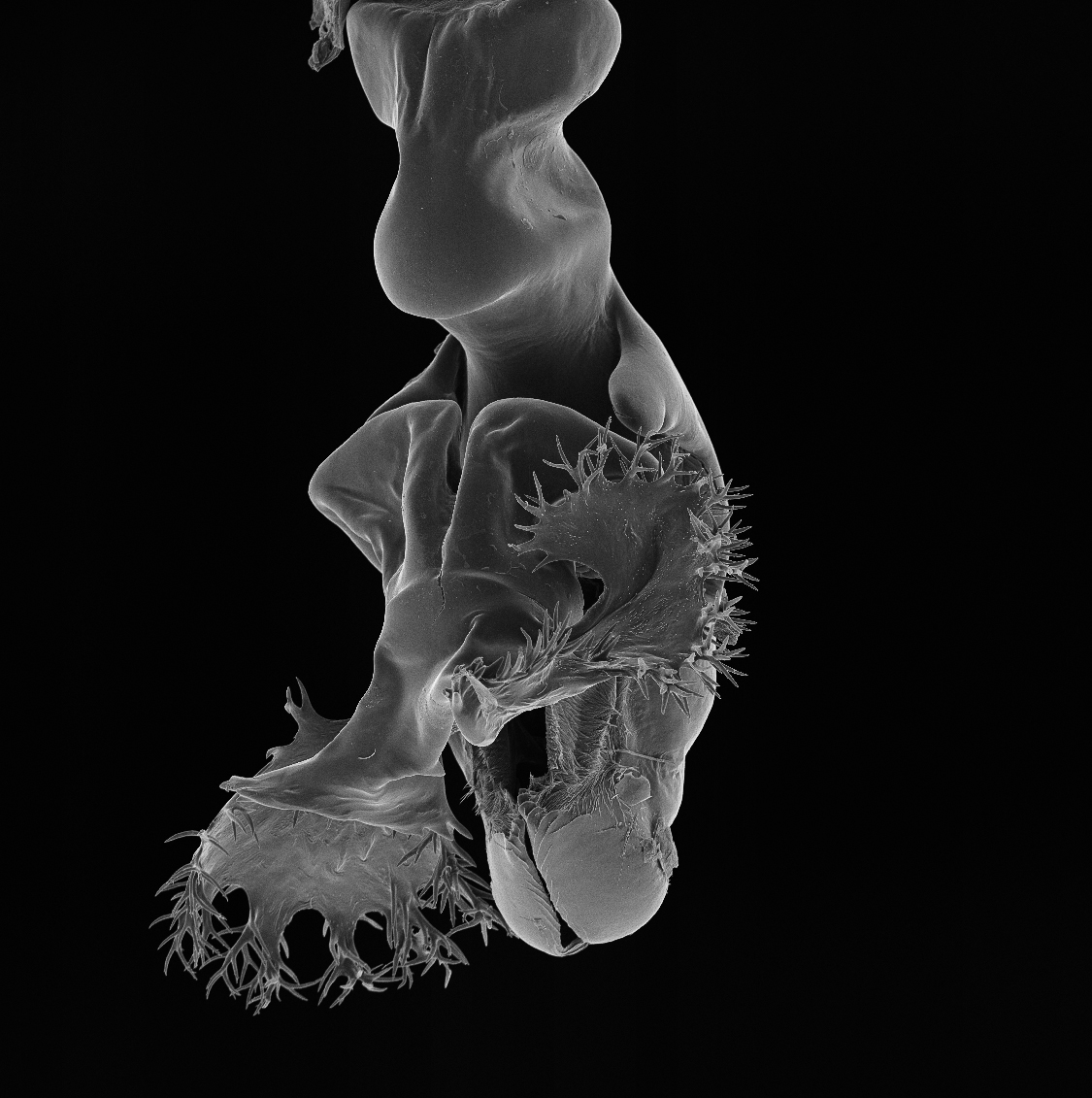

Supplement: Supplementary file 2 — rSEM illustrating the distiphallus of Oxysarcodexia (Xylocamptopsis) fringidea (Curran & Walley) (Sarcophagidae, Diptera); web-published using Magic 360TM script files. Click and drag to rotate the rSEM and point click to open and close the magnification tool. (doi: 10.3897/zookeys.328.5768.app2) File format: Hypertext Markup Document, archived (zip). [file ZooKeys-328-047-s002.zip › Fig S4 - magic360/fringidea/fringidea-lg-02.jpg]

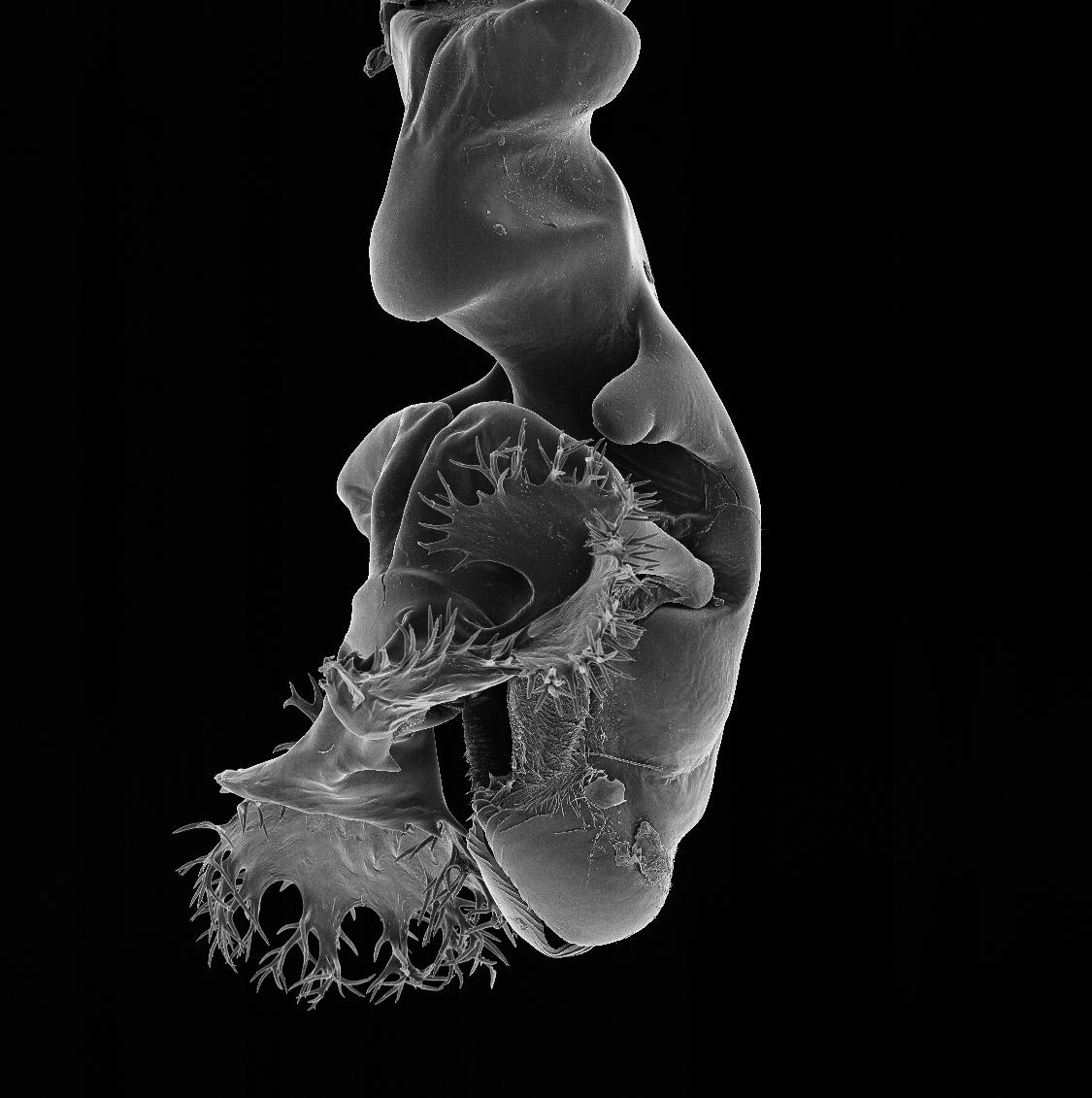

Supplement: Supplementary file 2 — rSEM illustrating the distiphallus of Oxysarcodexia (Xylocamptopsis) fringidea (Curran & Walley) (Sarcophagidae, Diptera); web-published using Magic 360TM script files. Click and drag to rotate the rSEM and point click to open and close the magnification tool. (doi: 10.3897/zookeys.328.5768.app2) File format: Hypertext Markup Document, archived (zip). [file ZooKeys-328-047-s002.zip › Fig S4 - magic360/fringidea/fringidea-lg-03.jpg]

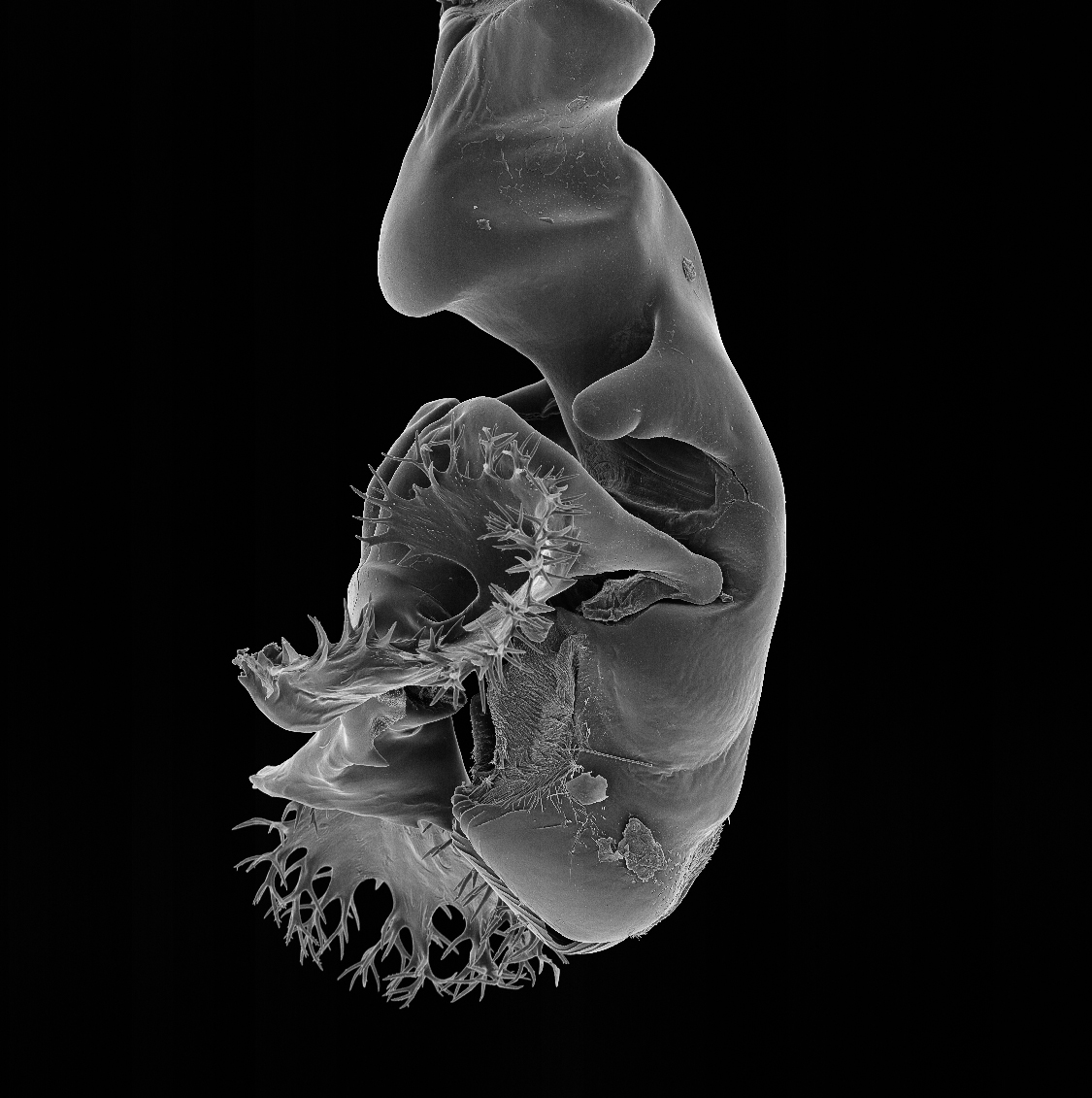

Supplement: Supplementary file 2 — rSEM illustrating the distiphallus of Oxysarcodexia (Xylocamptopsis) fringidea (Curran & Walley) (Sarcophagidae, Diptera); web-published using Magic 360TM script files. Click and drag to rotate the rSEM and point click to open and close the magnification tool. (doi: 10.3897/zookeys.328.5768.app2) File format: Hypertext Markup Document, archived (zip). [file ZooKeys-328-047-s002.zip › Fig S4 - magic360/fringidea/fringidea-lg-04.jpg]

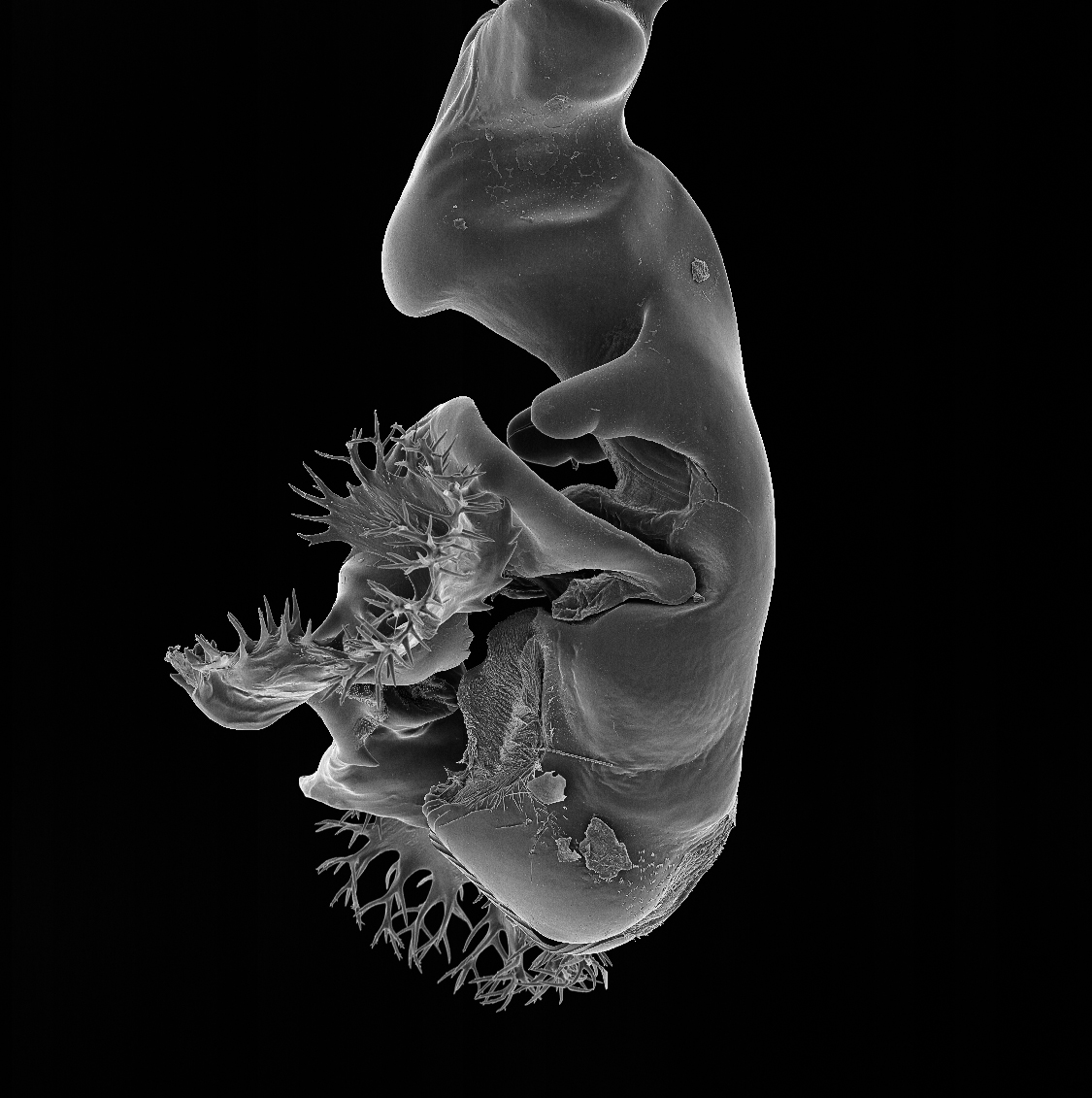

Supplement: Supplementary file 2 — rSEM illustrating the distiphallus of Oxysarcodexia (Xylocamptopsis) fringidea (Curran & Walley) (Sarcophagidae, Diptera); web-published using Magic 360TM script files. Click and drag to rotate the rSEM and point click to open and close the magnification tool. (doi: 10.3897/zookeys.328.5768.app2) File format: Hypertext Markup Document, archived (zip). [file ZooKeys-328-047-s002.zip › Fig S4 - magic360/fringidea/fringidea-lg-05.jpg]

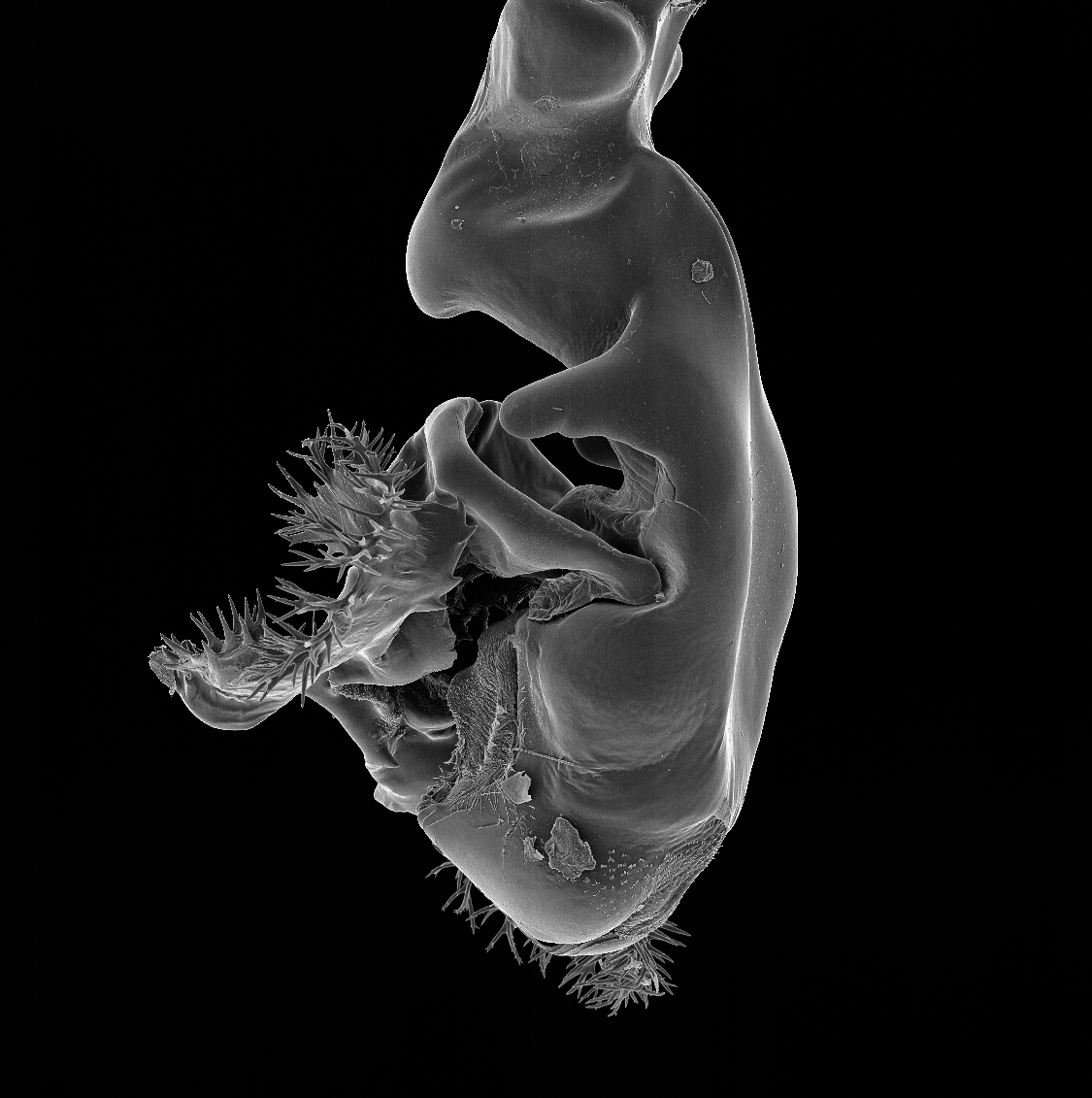

Supplement: Supplementary file 2 — rSEM illustrating the distiphallus of Oxysarcodexia (Xylocamptopsis) fringidea (Curran & Walley) (Sarcophagidae, Diptera); web-published using Magic 360TM script files. Click and drag to rotate the rSEM and point click to open and close the magnification tool. (doi: 10.3897/zookeys.328.5768.app2) File format: Hypertext Markup Document, archived (zip). [file ZooKeys-328-047-s002.zip › Fig S4 - magic360/fringidea/fringidea-lg-06.jpg]

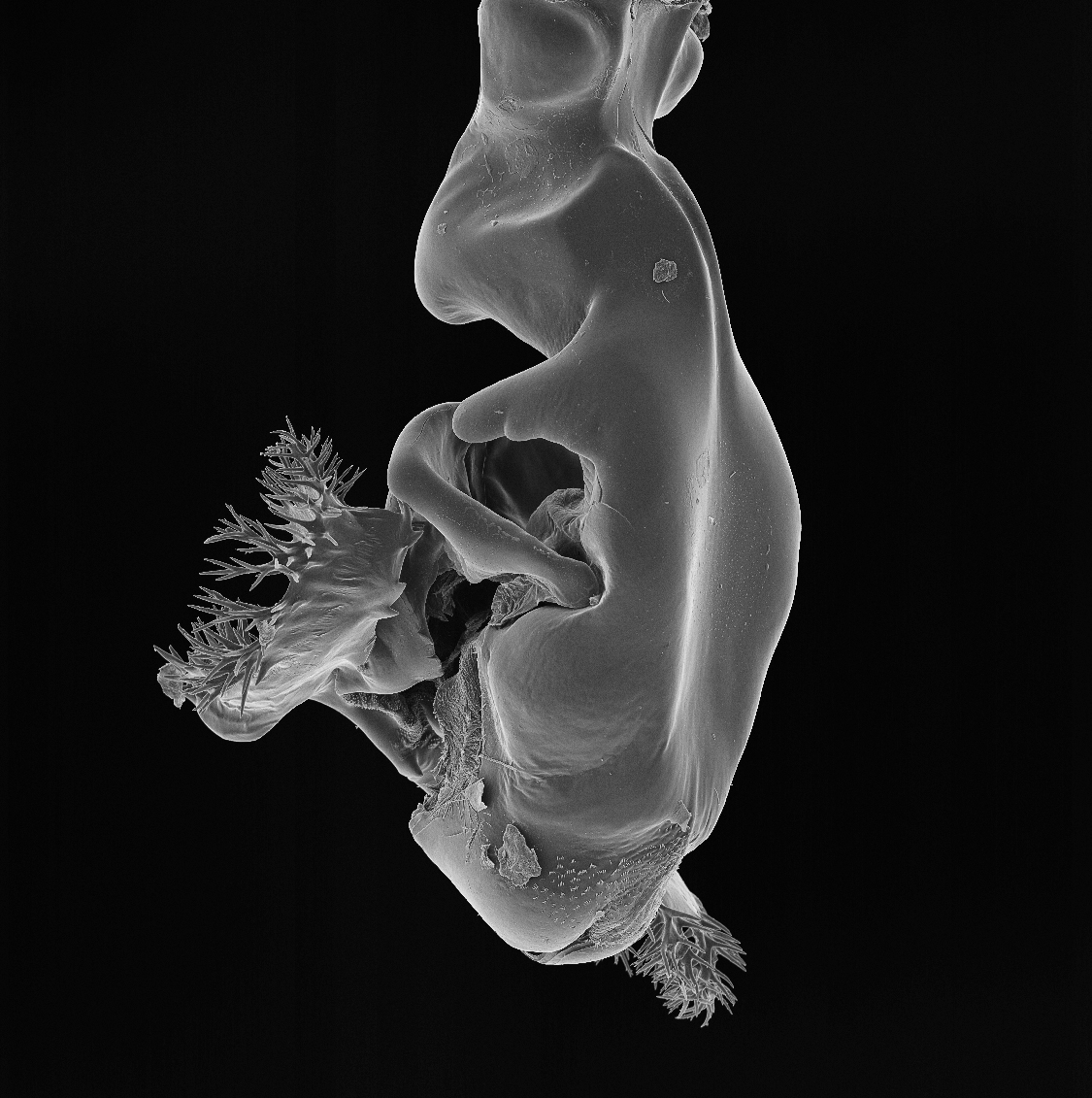

Supplement: Supplementary file 2 — rSEM illustrating the distiphallus of Oxysarcodexia (Xylocamptopsis) fringidea (Curran & Walley) (Sarcophagidae, Diptera); web-published using Magic 360TM script files. Click and drag to rotate the rSEM and point click to open and close the magnification tool. (doi: 10.3897/zookeys.328.5768.app2) File format: Hypertext Markup Document, archived (zip). [file ZooKeys-328-047-s002.zip › Fig S4 - magic360/fringidea/fringidea-lg-07.jpg]

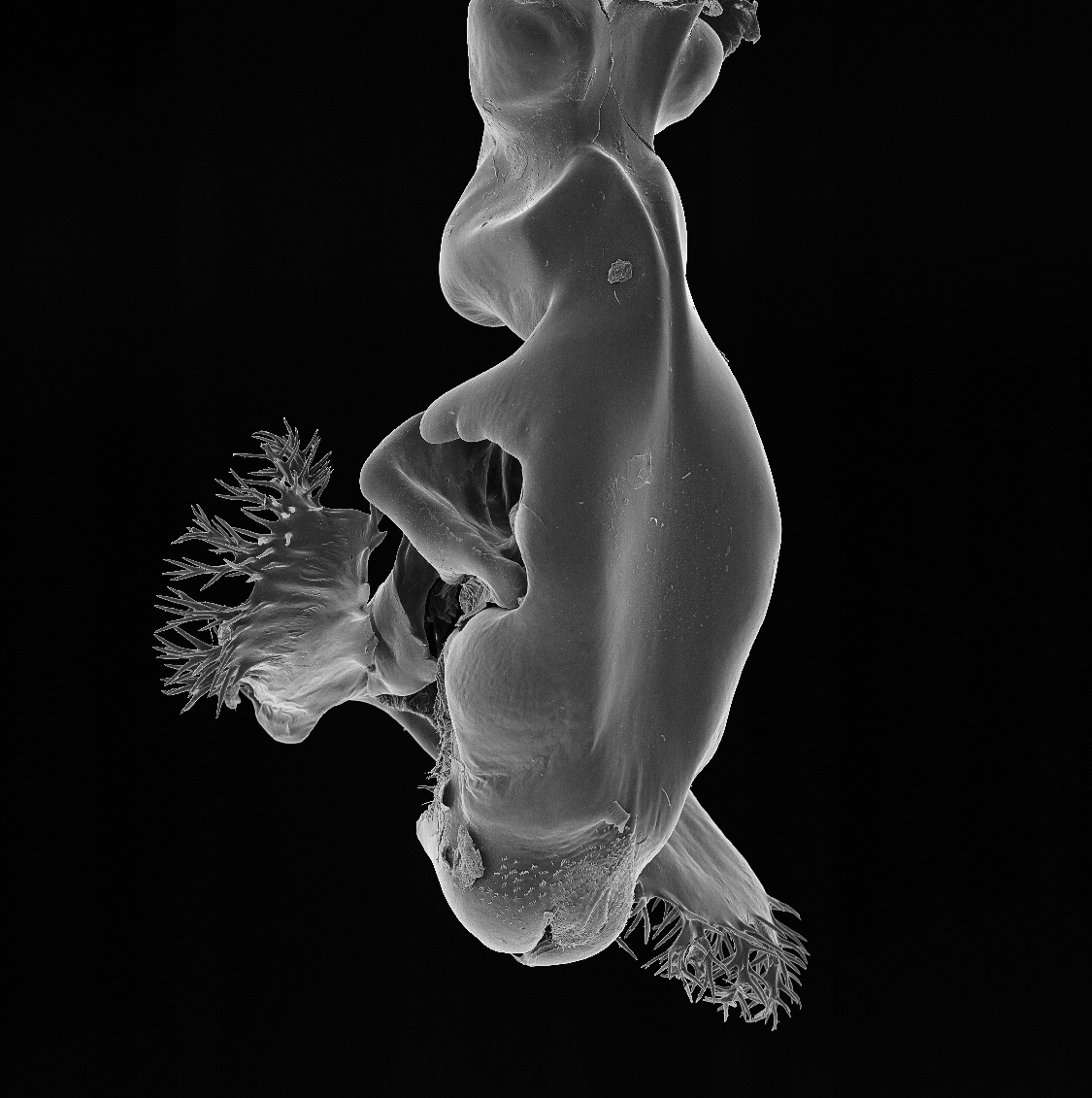

Supplement: Supplementary file 2 — rSEM illustrating the distiphallus of Oxysarcodexia (Xylocamptopsis) fringidea (Curran & Walley) (Sarcophagidae, Diptera); web-published using Magic 360TM script files. Click and drag to rotate the rSEM and point click to open and close the magnification tool. (doi: 10.3897/zookeys.328.5768.app2) File format: Hypertext Markup Document, archived (zip). [file ZooKeys-328-047-s002.zip › Fig S4 - magic360/fringidea/fringidea-lg-08.jpg]

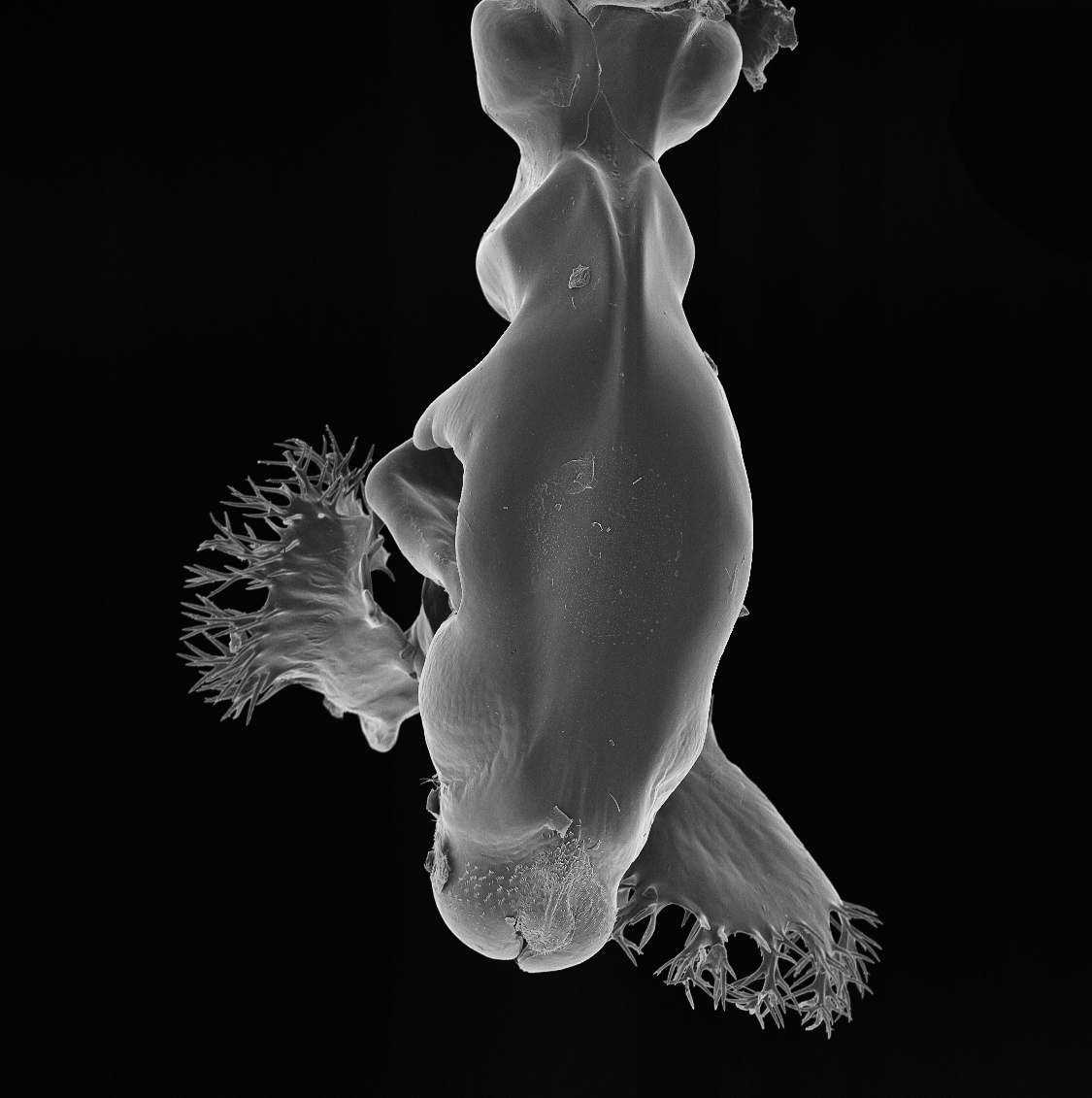

Supplement: Supplementary file 2 — rSEM illustrating the distiphallus of Oxysarcodexia (Xylocamptopsis) fringidea (Curran & Walley) (Sarcophagidae, Diptera); web-published using Magic 360TM script files. Click and drag to rotate the rSEM and point click to open and close the magnification tool. (doi: 10.3897/zookeys.328.5768.app2) File format: Hypertext Markup Document, archived (zip). [file ZooKeys-328-047-s002.zip › Fig S4 - magic360/fringidea/fringidea-lg-09.jpg]

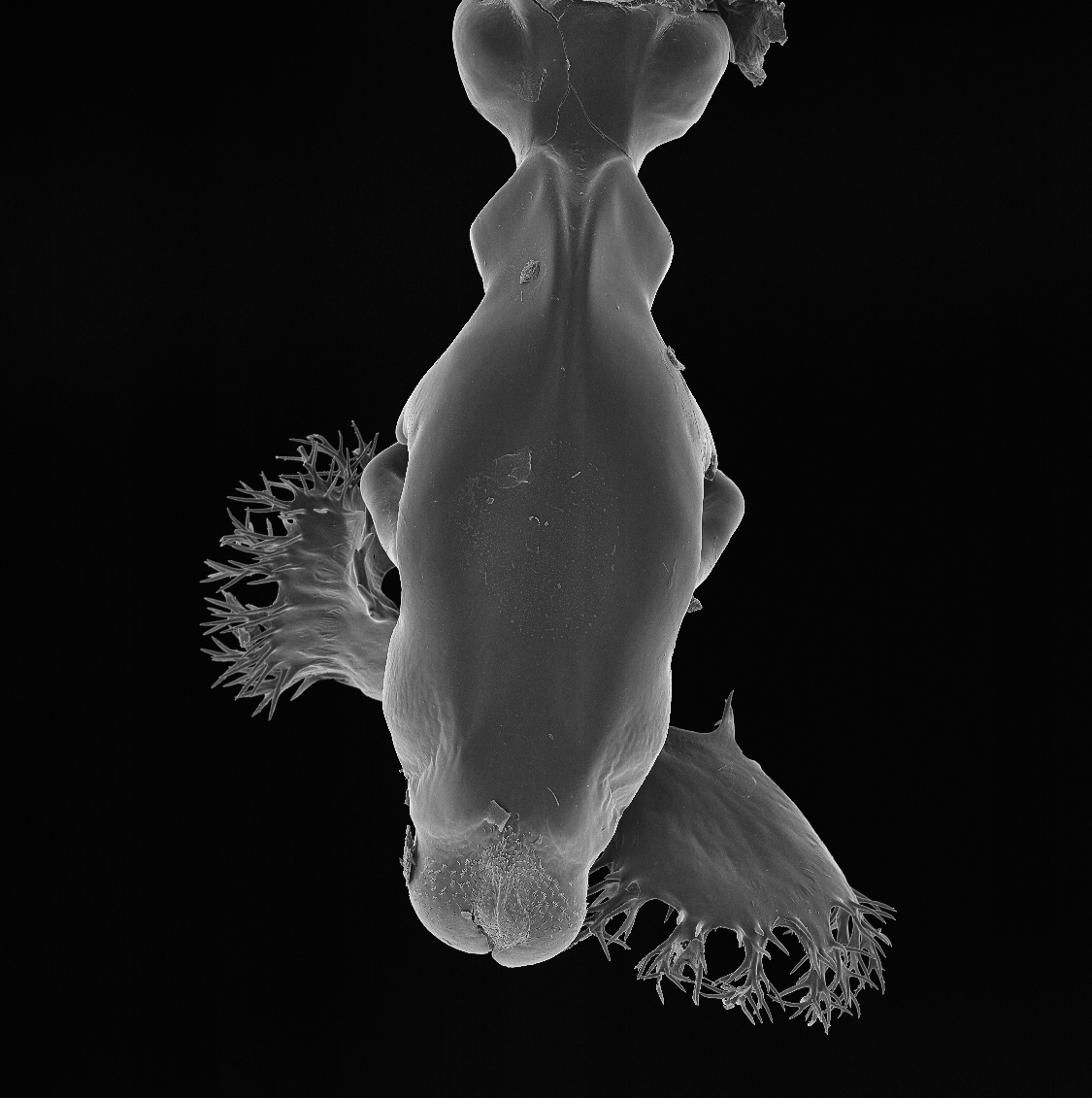

Supplement: Supplementary file 2 — rSEM illustrating the distiphallus of Oxysarcodexia (Xylocamptopsis) fringidea (Curran & Walley) (Sarcophagidae, Diptera); web-published using Magic 360TM script files. Click and drag to rotate the rSEM and point click to open and close the magnification tool. (doi: 10.3897/zookeys.328.5768.app2) File format: Hypertext Markup Document, archived (zip). [file ZooKeys-328-047-s002.zip › Fig S4 - magic360/fringidea/fringidea-lg-10.jpg]

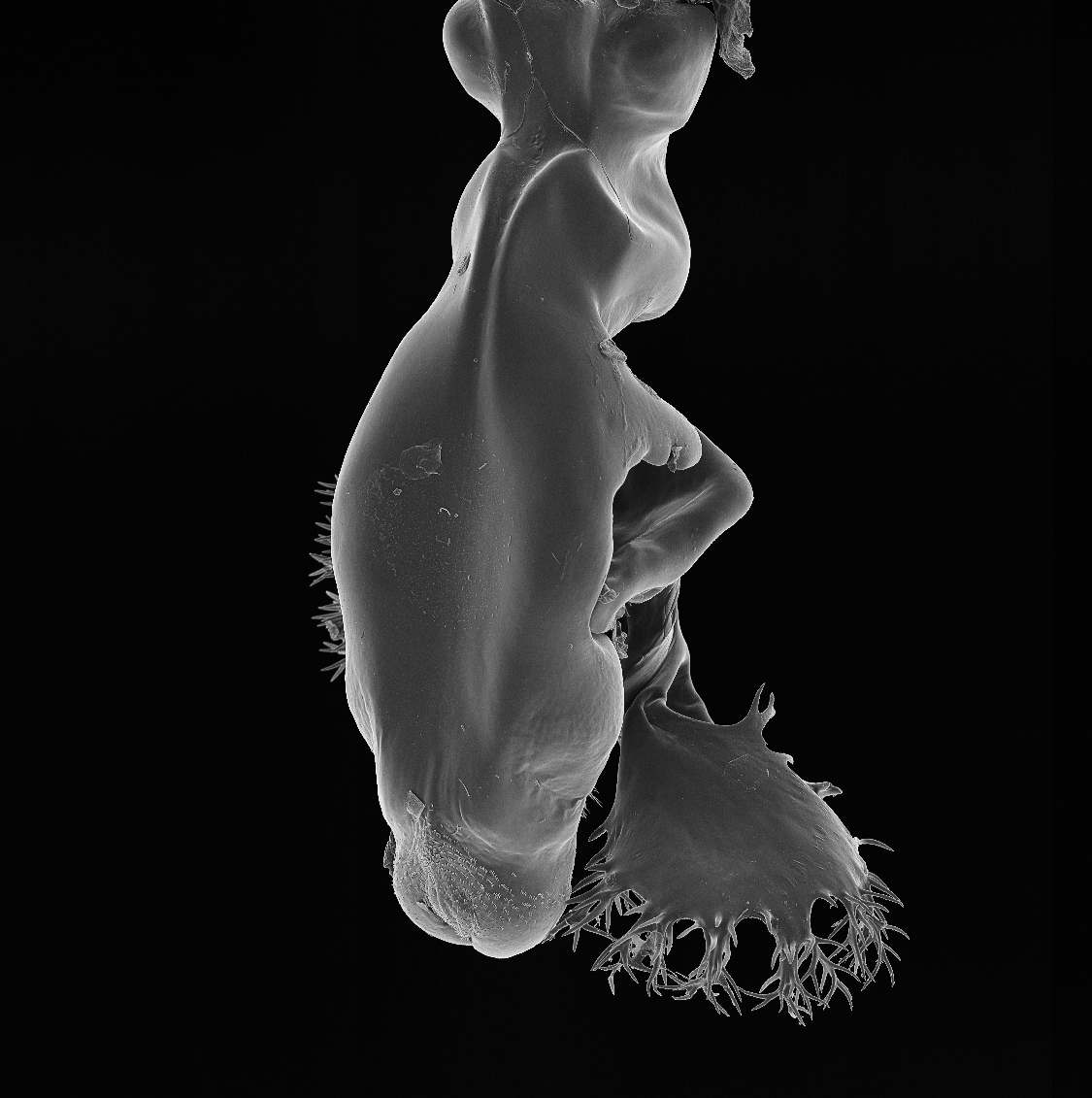

Supplement: Supplementary file 2 — rSEM illustrating the distiphallus of Oxysarcodexia (Xylocamptopsis) fringidea (Curran & Walley) (Sarcophagidae, Diptera); web-published using Magic 360TM script files. Click and drag to rotate the rSEM and point click to open and close the magnification tool. (doi: 10.3897/zookeys.328.5768.app2) File format: Hypertext Markup Document, archived (zip). [file ZooKeys-328-047-s002.zip › Fig S4 - magic360/fringidea/fringidea-lg-11.jpg]

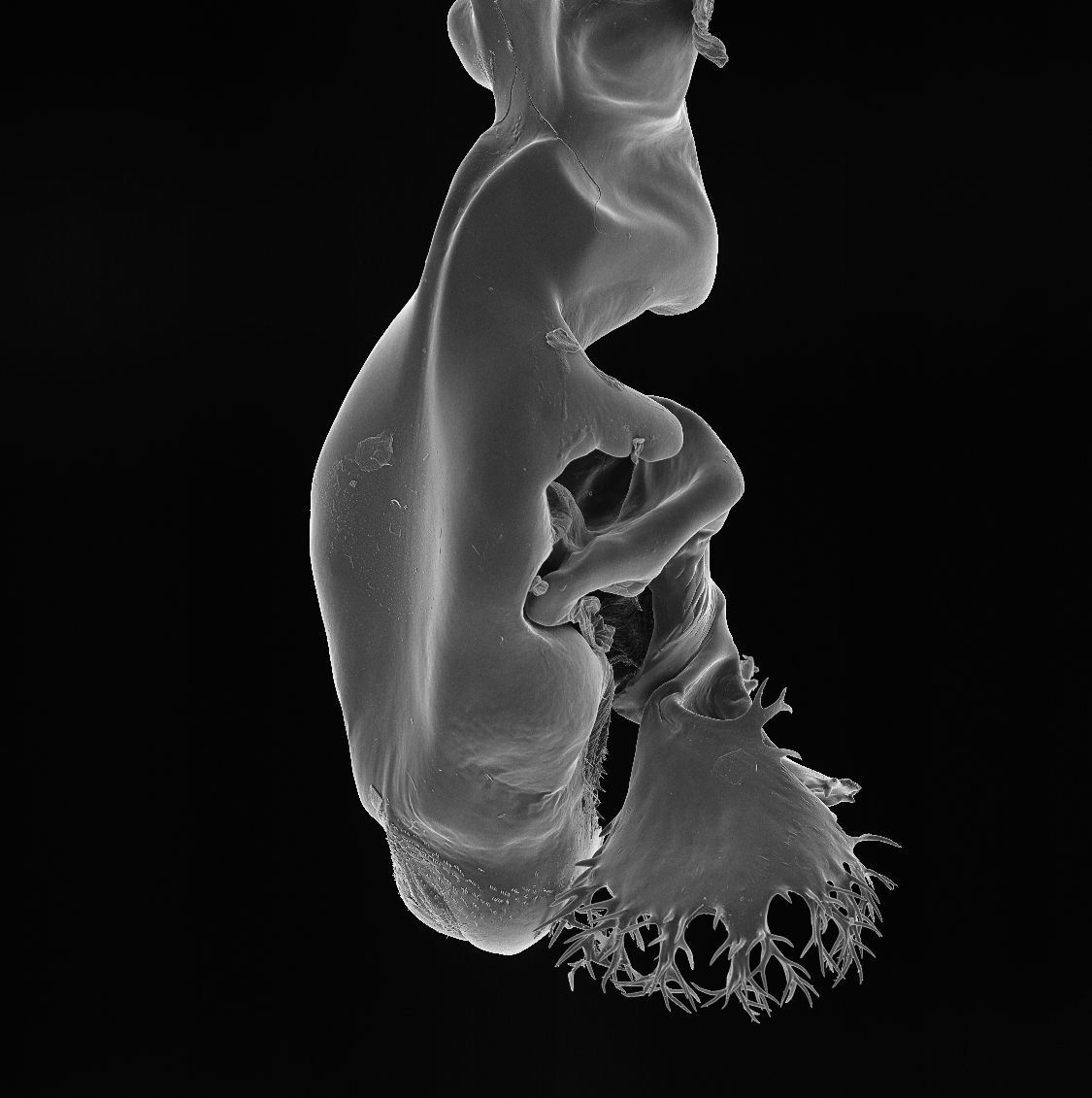

Supplement: Supplementary file 2 — rSEM illustrating the distiphallus of Oxysarcodexia (Xylocamptopsis) fringidea (Curran & Walley) (Sarcophagidae, Diptera); web-published using Magic 360TM script files. Click and drag to rotate the rSEM and point click to open and close the magnification tool. (doi: 10.3897/zookeys.328.5768.app2) File format: Hypertext Markup Document, archived (zip). [file ZooKeys-328-047-s002.zip › Fig S4 - magic360/fringidea/fringidea-lg-12.jpg]

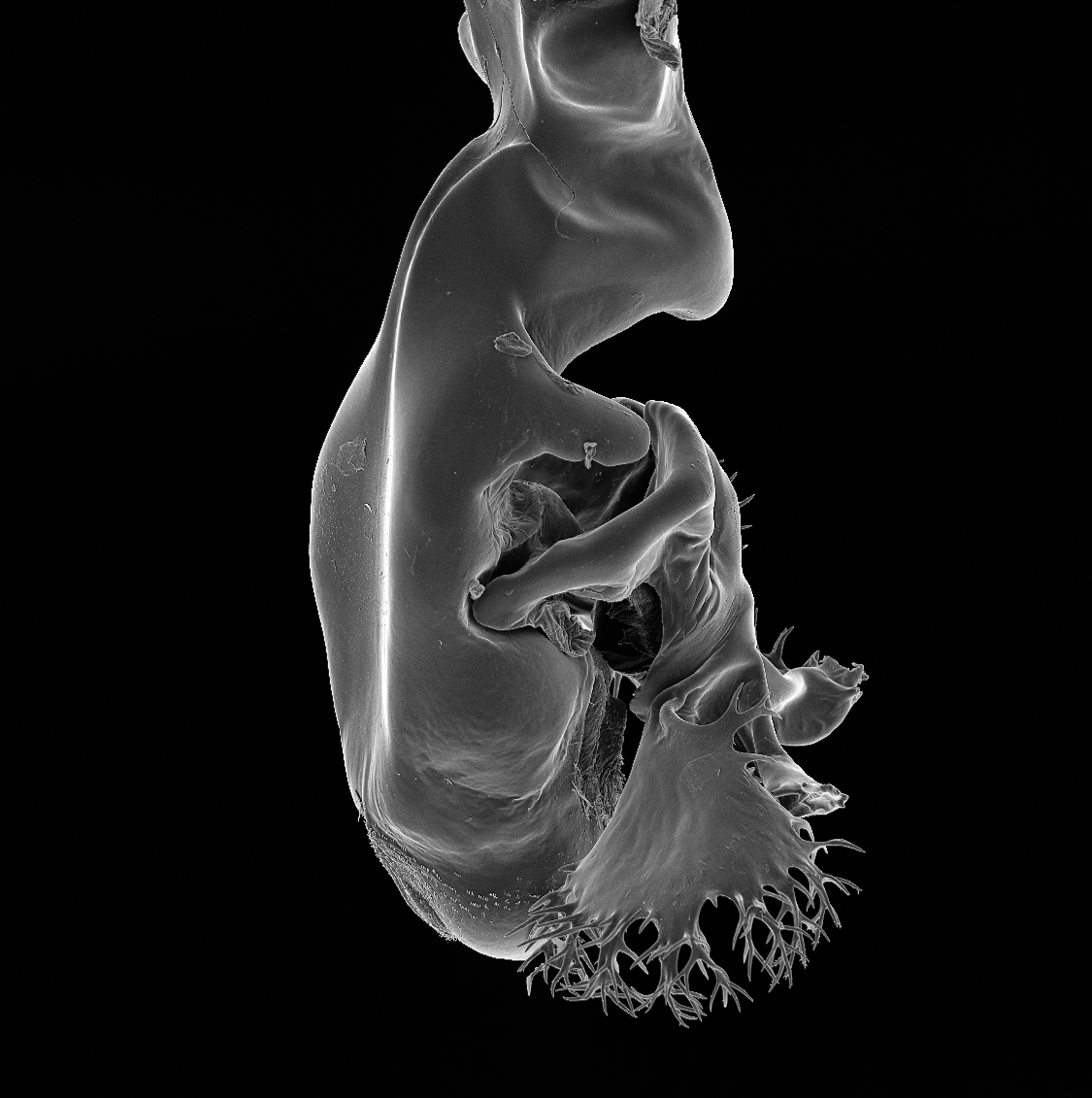

Supplement: Supplementary file 2 — rSEM illustrating the distiphallus of Oxysarcodexia (Xylocamptopsis) fringidea (Curran & Walley) (Sarcophagidae, Diptera); web-published using Magic 360TM script files. Click and drag to rotate the rSEM and point click to open and close the magnification tool. (doi: 10.3897/zookeys.328.5768.app2) File format: Hypertext Markup Document, archived (zip). [file ZooKeys-328-047-s002.zip › Fig S4 - magic360/fringidea/fringidea-lg-13.jpg]

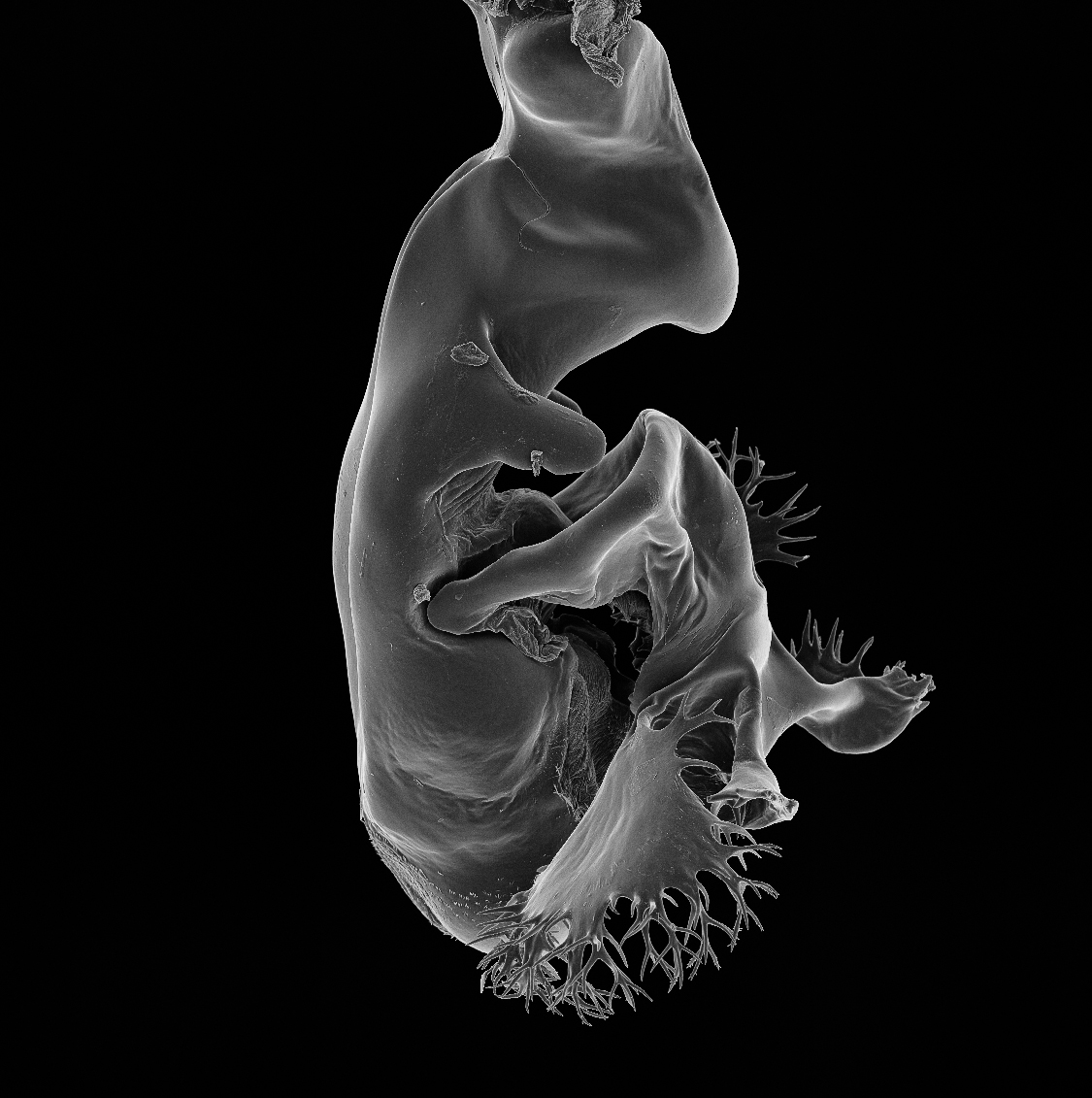

Supplement: Supplementary file 2 — rSEM illustrating the distiphallus of Oxysarcodexia (Xylocamptopsis) fringidea (Curran & Walley) (Sarcophagidae, Diptera); web-published using Magic 360TM script files. Click and drag to rotate the rSEM and point click to open and close the magnification tool. (doi: 10.3897/zookeys.328.5768.app2) File format: Hypertext Markup Document, archived (zip). [file ZooKeys-328-047-s002.zip › Fig S4 - magic360/fringidea/fringidea-lg-14.jpg]

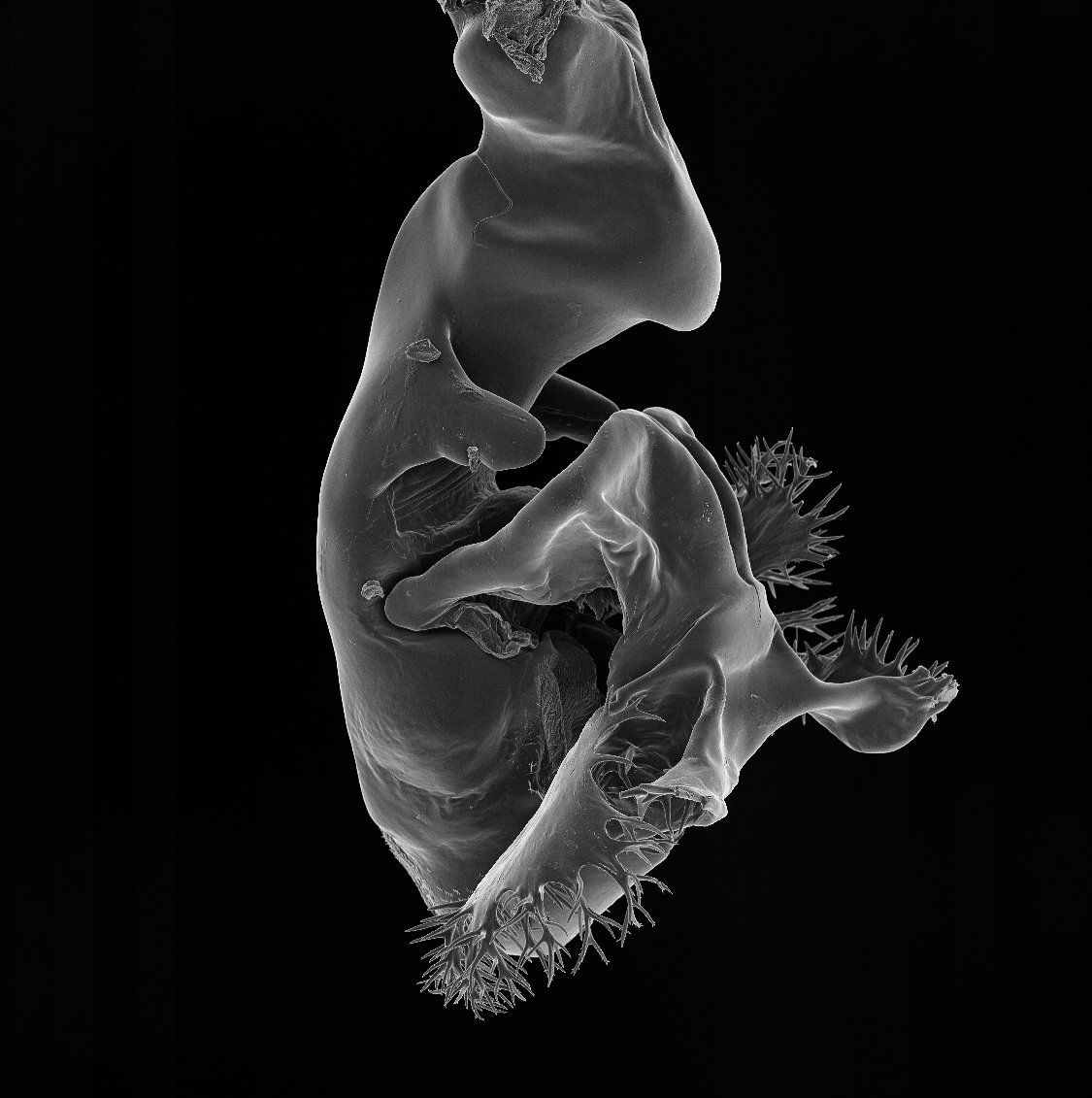

Supplement: Supplementary file 2 — rSEM illustrating the distiphallus of Oxysarcodexia (Xylocamptopsis) fringidea (Curran & Walley) (Sarcophagidae, Diptera); web-published using Magic 360TM script files. Click and drag to rotate the rSEM and point click to open and close the magnification tool. (doi: 10.3897/zookeys.328.5768.app2) File format: Hypertext Markup Document, archived (zip). [file ZooKeys-328-047-s002.zip › Fig S4 - magic360/fringidea/fringidea-lg-15.jpg]

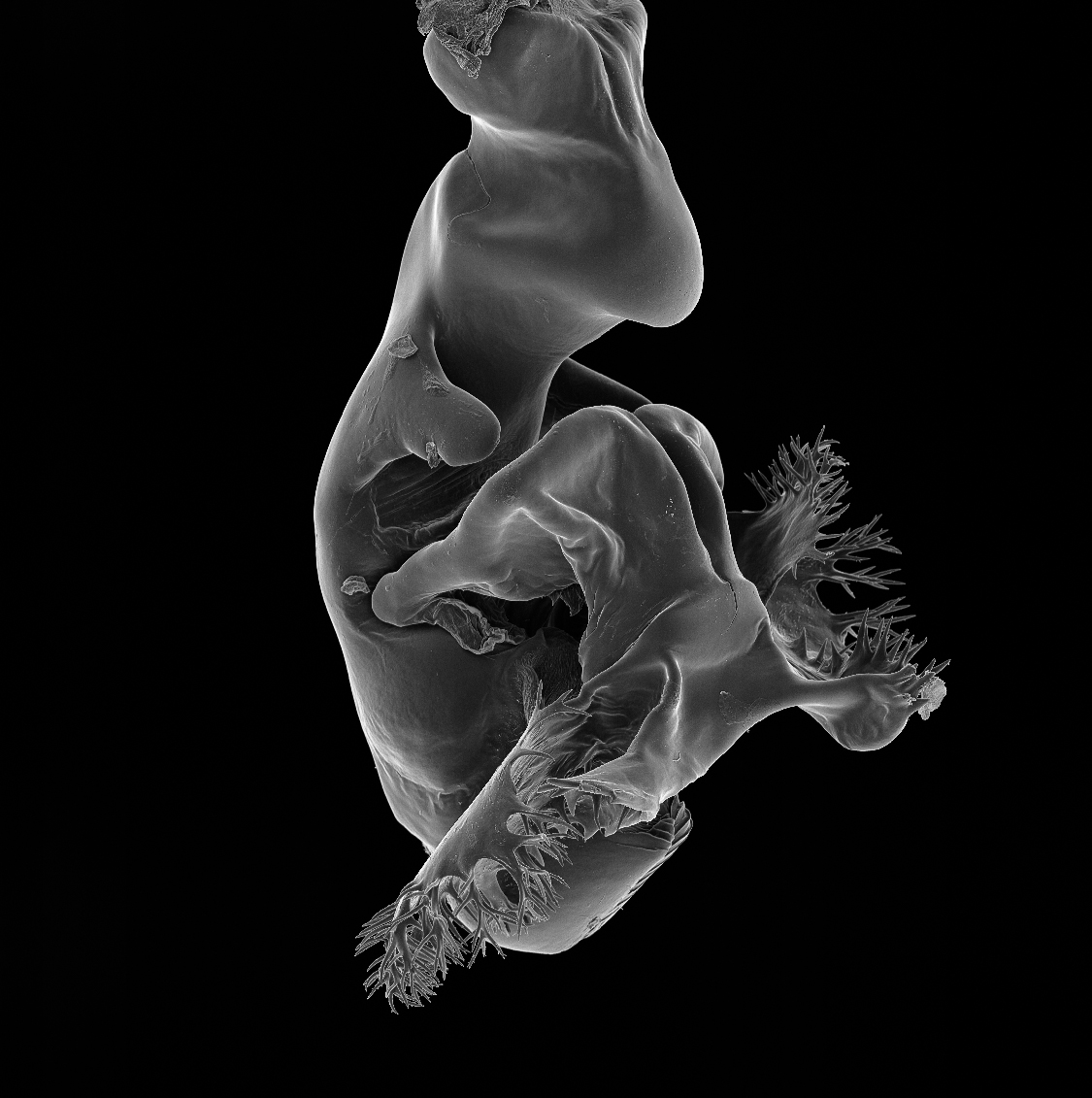

Supplement: Supplementary file 2 — rSEM illustrating the distiphallus of Oxysarcodexia (Xylocamptopsis) fringidea (Curran & Walley) (Sarcophagidae, Diptera); web-published using Magic 360TM script files. Click and drag to rotate the rSEM and point click to open and close the magnification tool. (doi: 10.3897/zookeys.328.5768.app2) File format: Hypertext Markup Document, archived (zip). [file ZooKeys-328-047-s002.zip › Fig S4 - magic360/fringidea/fringidea-lg-16.jpg]

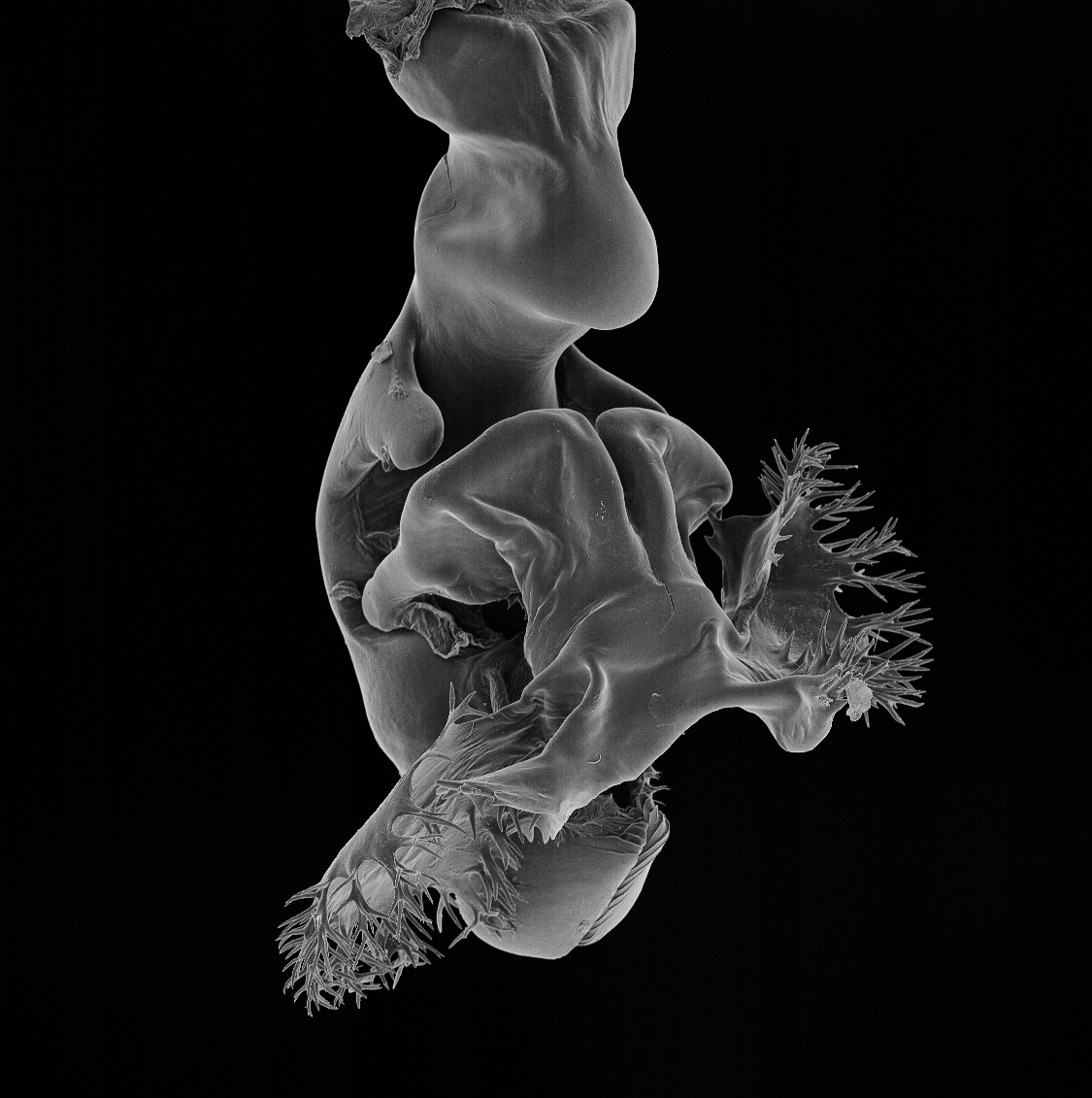

Supplement: Supplementary file 2 — rSEM illustrating the distiphallus of Oxysarcodexia (Xylocamptopsis) fringidea (Curran & Walley) (Sarcophagidae, Diptera); web-published using Magic 360TM script files. Click and drag to rotate the rSEM and point click to open and close the magnification tool. (doi: 10.3897/zookeys.328.5768.app2) File format: Hypertext Markup Document, archived (zip). [file ZooKeys-328-047-s002.zip › Fig S4 - magic360/fringidea/fringidea-lg-17.jpg]

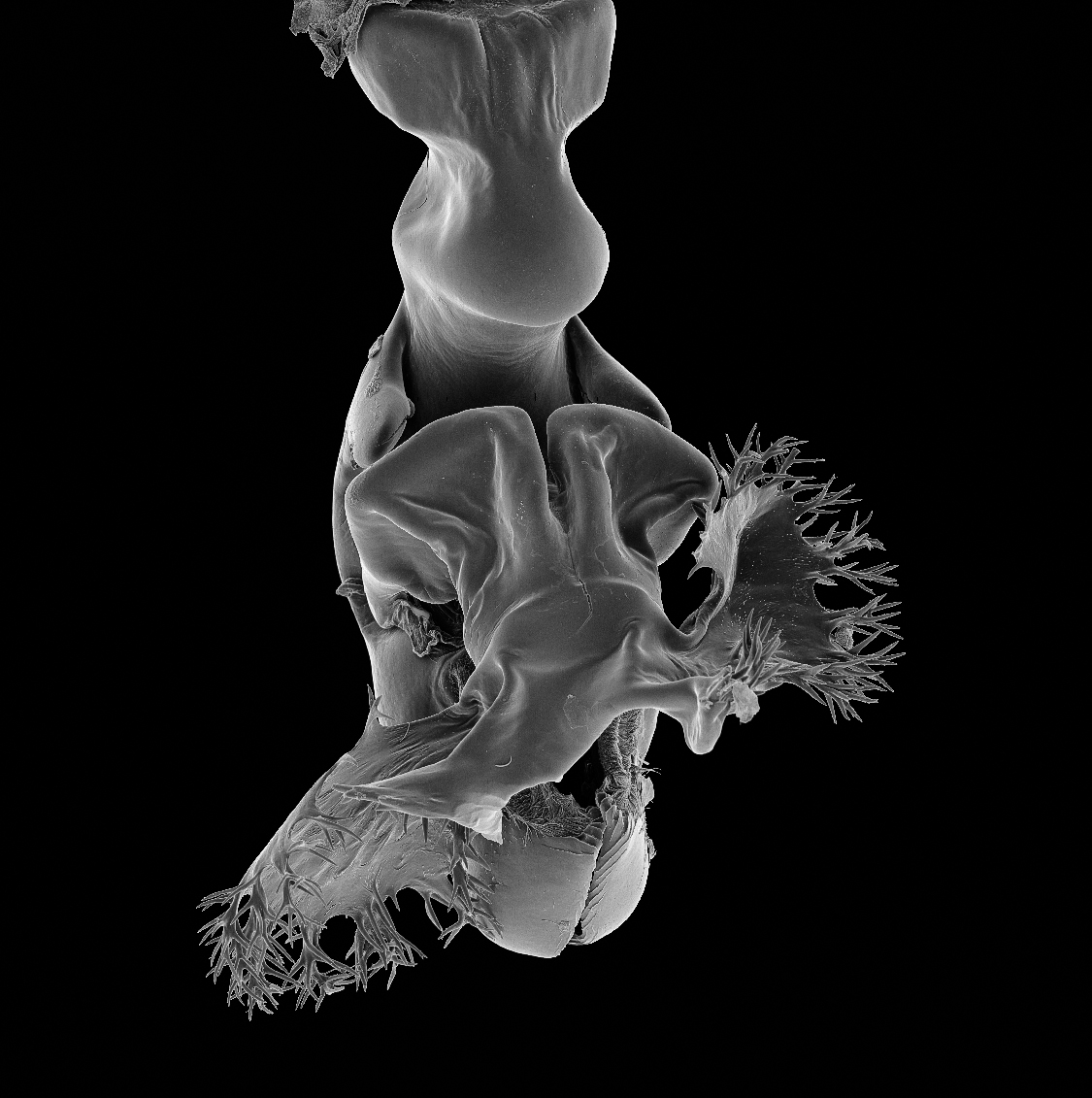

Supplement: Supplementary file 2 — rSEM illustrating the distiphallus of Oxysarcodexia (Xylocamptopsis) fringidea (Curran & Walley) (Sarcophagidae, Diptera); web-published using Magic 360TM script files. Click and drag to rotate the rSEM and point click to open and close the magnification tool. (doi: 10.3897/zookeys.328.5768.app2) File format: Hypertext Markup Document, archived (zip). [file ZooKeys-328-047-s002.zip › Fig S4 - magic360/fringidea/fringidea-lg-18.jpg]

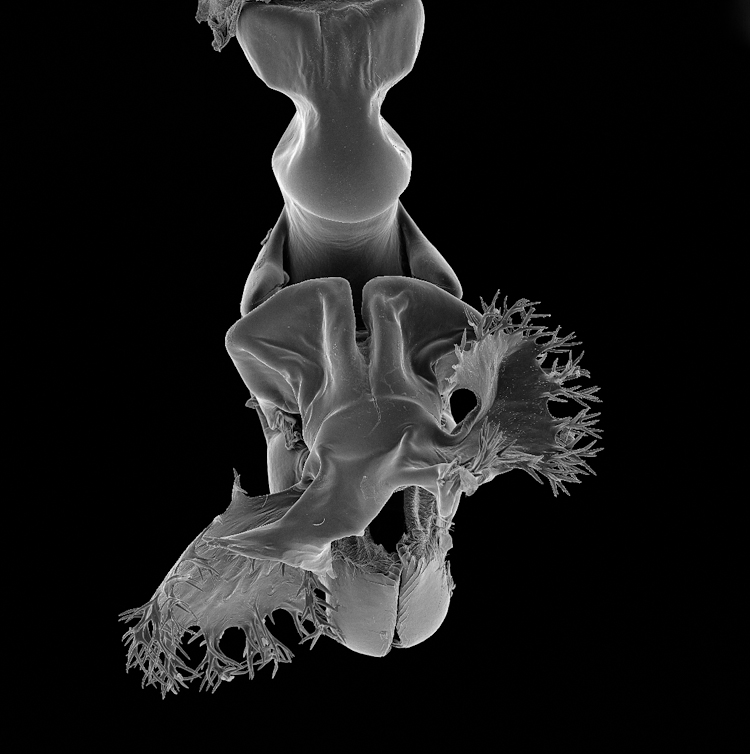

Supplement: Supplementary file 2 — rSEM illustrating the distiphallus of Oxysarcodexia (Xylocamptopsis) fringidea (Curran & Walley) (Sarcophagidae, Diptera); web-published using Magic 360TM script files. Click and drag to rotate the rSEM and point click to open and close the magnification tool. (doi: 10.3897/zookeys.328.5768.app2) File format: Hypertext Markup Document, archived (zip). [file ZooKeys-328-047-s002.zip › Fig S4 - magic360/fringidea/fringidea-sm-01.jpg]

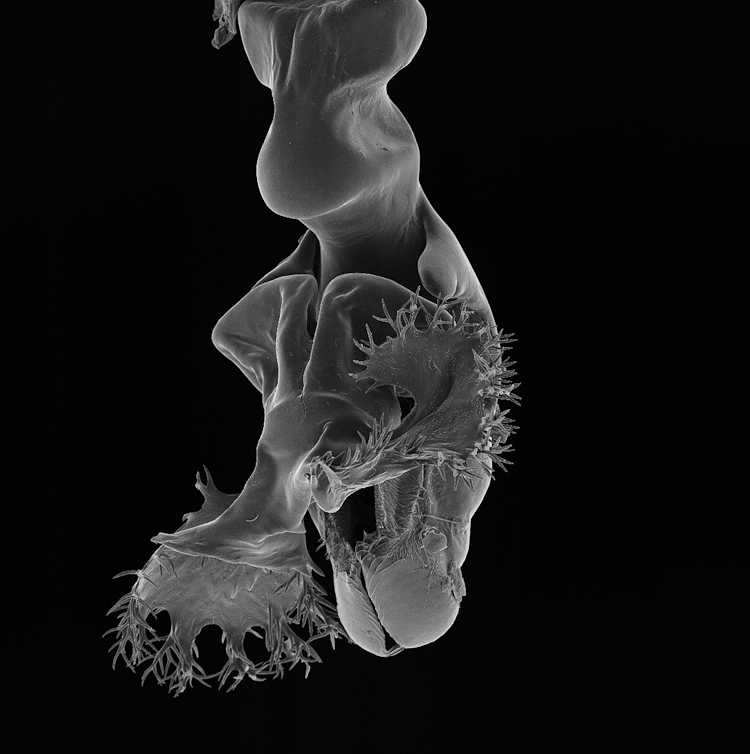

Supplement: Supplementary file 2 — rSEM illustrating the distiphallus of Oxysarcodexia (Xylocamptopsis) fringidea (Curran & Walley) (Sarcophagidae, Diptera); web-published using Magic 360TM script files. Click and drag to rotate the rSEM and point click to open and close the magnification tool. (doi: 10.3897/zookeys.328.5768.app2) File format: Hypertext Markup Document, archived (zip). [file ZooKeys-328-047-s002.zip › Fig S4 - magic360/fringidea/fringidea-sm-02.jpg]

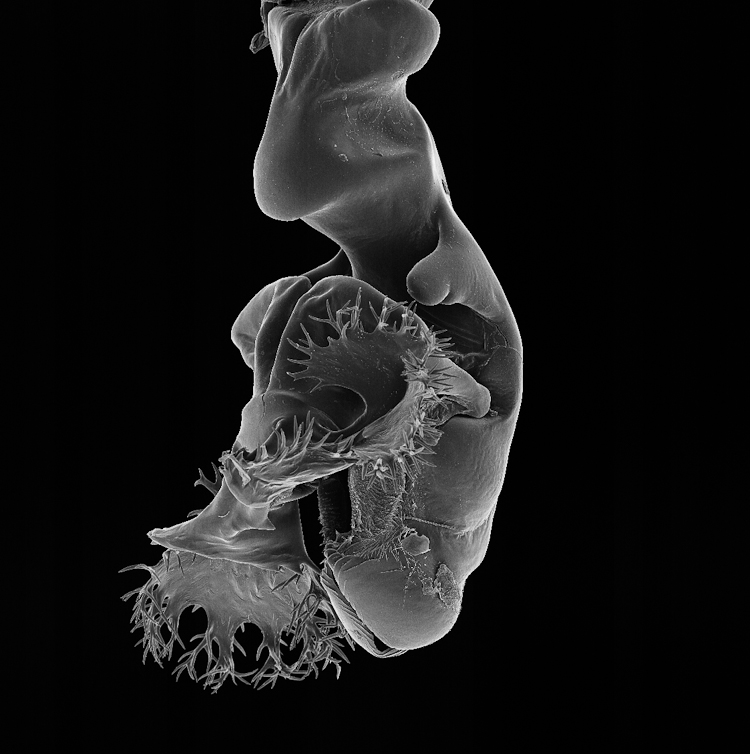

Supplement: Supplementary file 2 — rSEM illustrating the distiphallus of Oxysarcodexia (Xylocamptopsis) fringidea (Curran & Walley) (Sarcophagidae, Diptera); web-published using Magic 360TM script files. Click and drag to rotate the rSEM and point click to open and close the magnification tool. (doi: 10.3897/zookeys.328.5768.app2) File format: Hypertext Markup Document, archived (zip). [file ZooKeys-328-047-s002.zip › Fig S4 - magic360/fringidea/fringidea-sm-03.jpg]

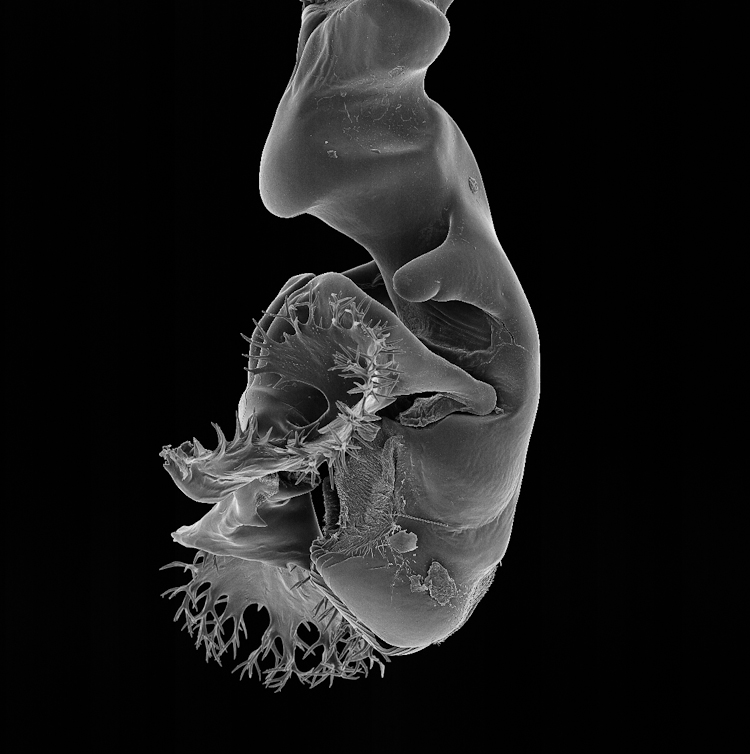

Supplement: Supplementary file 2 — rSEM illustrating the distiphallus of Oxysarcodexia (Xylocamptopsis) fringidea (Curran & Walley) (Sarcophagidae, Diptera); web-published using Magic 360TM script files. Click and drag to rotate the rSEM and point click to open and close the magnification tool. (doi: 10.3897/zookeys.328.5768.app2) File format: Hypertext Markup Document, archived (zip). [file ZooKeys-328-047-s002.zip › Fig S4 - magic360/fringidea/fringidea-sm-04.jpg]

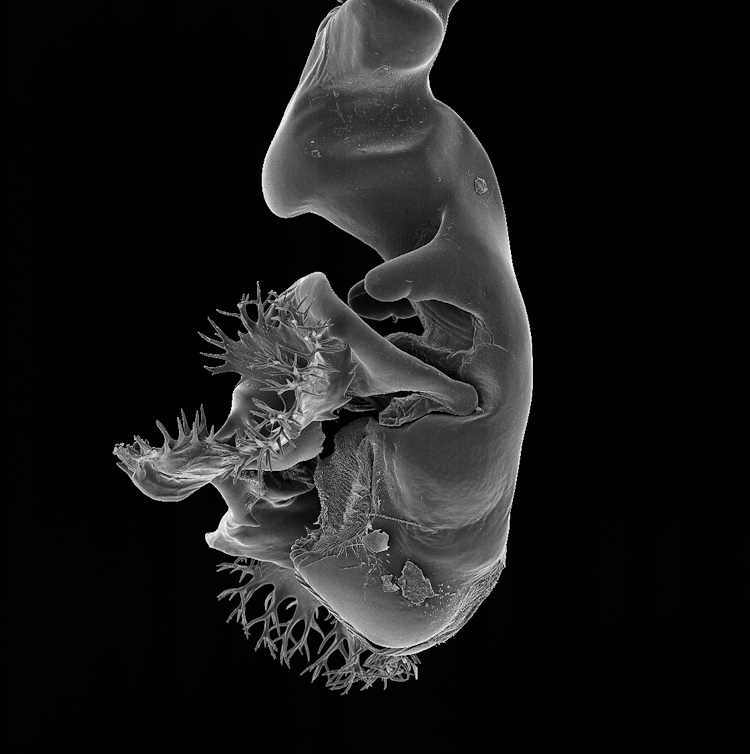

Supplement: Supplementary file 2 — rSEM illustrating the distiphallus of Oxysarcodexia (Xylocamptopsis) fringidea (Curran & Walley) (Sarcophagidae, Diptera); web-published using Magic 360TM script files. Click and drag to rotate the rSEM and point click to open and close the magnification tool. (doi: 10.3897/zookeys.328.5768.app2) File format: Hypertext Markup Document, archived (zip). [file ZooKeys-328-047-s002.zip › Fig S4 - magic360/fringidea/fringidea-sm-05.jpg]

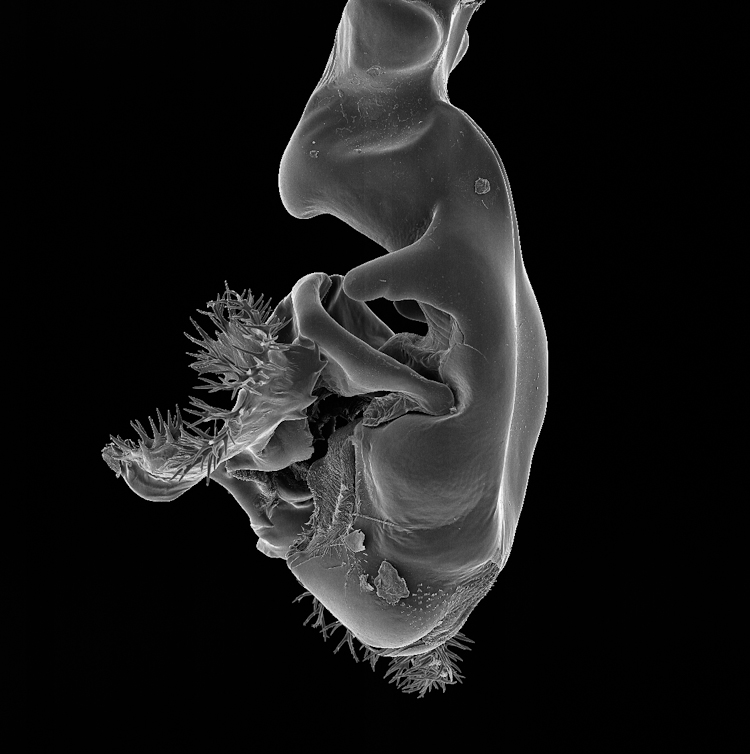

Supplement: Supplementary file 2 — rSEM illustrating the distiphallus of Oxysarcodexia (Xylocamptopsis) fringidea (Curran & Walley) (Sarcophagidae, Diptera); web-published using Magic 360TM script files. Click and drag to rotate the rSEM and point click to open and close the magnification tool. (doi: 10.3897/zookeys.328.5768.app2) File format: Hypertext Markup Document, archived (zip). [file ZooKeys-328-047-s002.zip › Fig S4 - magic360/fringidea/fringidea-sm-06.jpg]

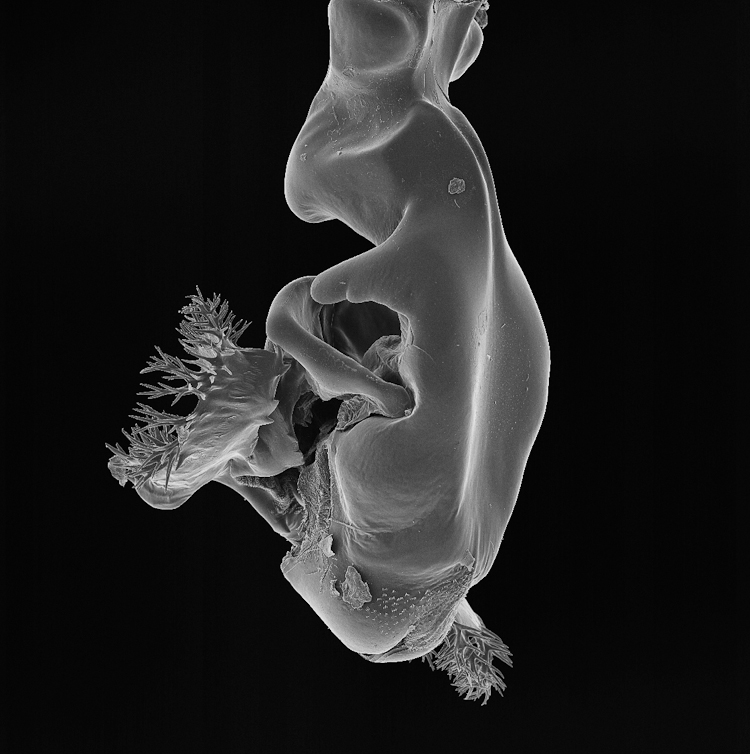

Supplement: Supplementary file 2 — rSEM illustrating the distiphallus of Oxysarcodexia (Xylocamptopsis) fringidea (Curran & Walley) (Sarcophagidae, Diptera); web-published using Magic 360TM script files. Click and drag to rotate the rSEM and point click to open and close the magnification tool. (doi: 10.3897/zookeys.328.5768.app2) File format: Hypertext Markup Document, archived (zip). [file ZooKeys-328-047-s002.zip › Fig S4 - magic360/fringidea/fringidea-sm-07.jpg]

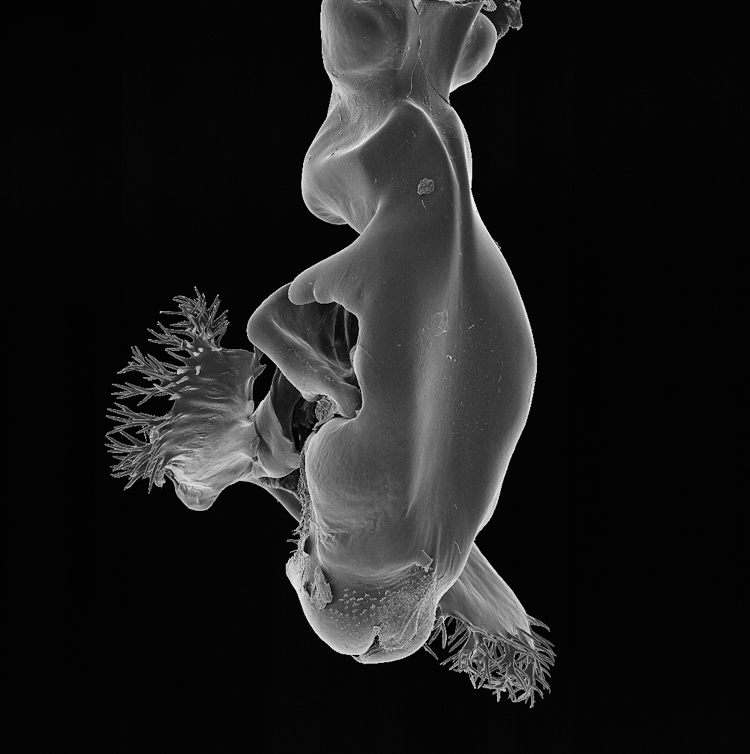

Supplement: Supplementary file 2 — rSEM illustrating the distiphallus of Oxysarcodexia (Xylocamptopsis) fringidea (Curran & Walley) (Sarcophagidae, Diptera); web-published using Magic 360TM script files. Click and drag to rotate the rSEM and point click to open and close the magnification tool. (doi: 10.3897/zookeys.328.5768.app2) File format: Hypertext Markup Document, archived (zip). [file ZooKeys-328-047-s002.zip › Fig S4 - magic360/fringidea/fringidea-sm-08.jpg]

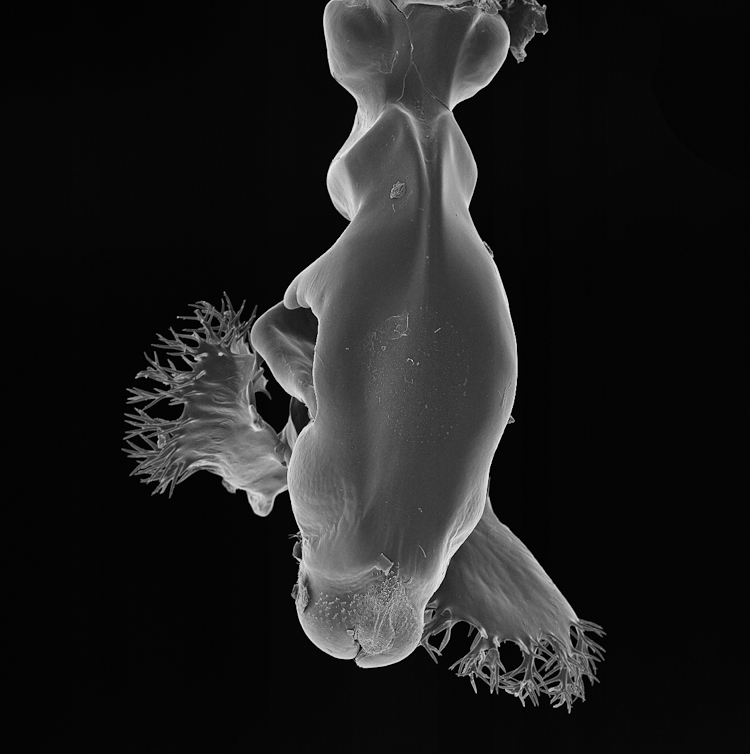

Supplement: Supplementary file 2 — rSEM illustrating the distiphallus of Oxysarcodexia (Xylocamptopsis) fringidea (Curran & Walley) (Sarcophagidae, Diptera); web-published using Magic 360TM script files. Click and drag to rotate the rSEM and point click to open and close the magnification tool. (doi: 10.3897/zookeys.328.5768.app2) File format: Hypertext Markup Document, archived (zip). [file ZooKeys-328-047-s002.zip › Fig S4 - magic360/fringidea/fringidea-sm-09.jpg]

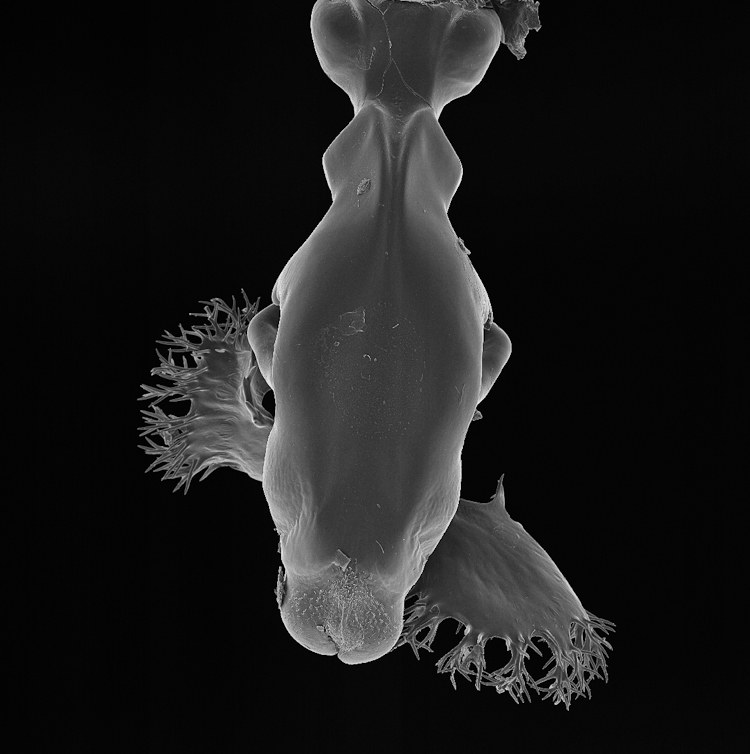

Supplement: Supplementary file 2 — rSEM illustrating the distiphallus of Oxysarcodexia (Xylocamptopsis) fringidea (Curran & Walley) (Sarcophagidae, Diptera); web-published using Magic 360TM script files. Click and drag to rotate the rSEM and point click to open and close the magnification tool. (doi: 10.3897/zookeys.328.5768.app2) File format: Hypertext Markup Document, archived (zip). [file ZooKeys-328-047-s002.zip › Fig S4 - magic360/fringidea/fringidea-sm-10.jpg]

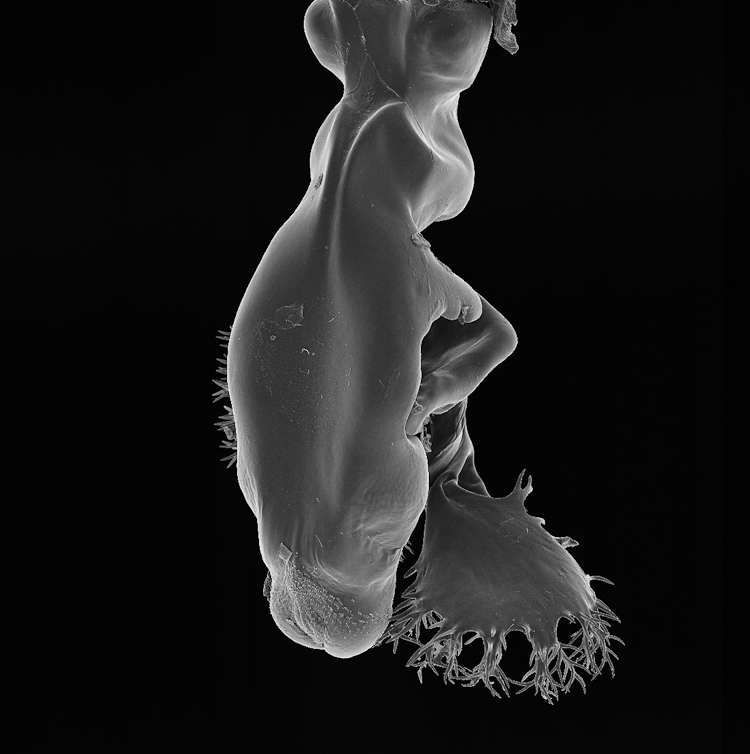

Supplement: Supplementary file 2 — rSEM illustrating the distiphallus of Oxysarcodexia (Xylocamptopsis) fringidea (Curran & Walley) (Sarcophagidae, Diptera); web-published using Magic 360TM script files. Click and drag to rotate the rSEM and point click to open and close the magnification tool. (doi: 10.3897/zookeys.328.5768.app2) File format: Hypertext Markup Document, archived (zip). [file ZooKeys-328-047-s002.zip › Fig S4 - magic360/fringidea/fringidea-sm-11.jpg]

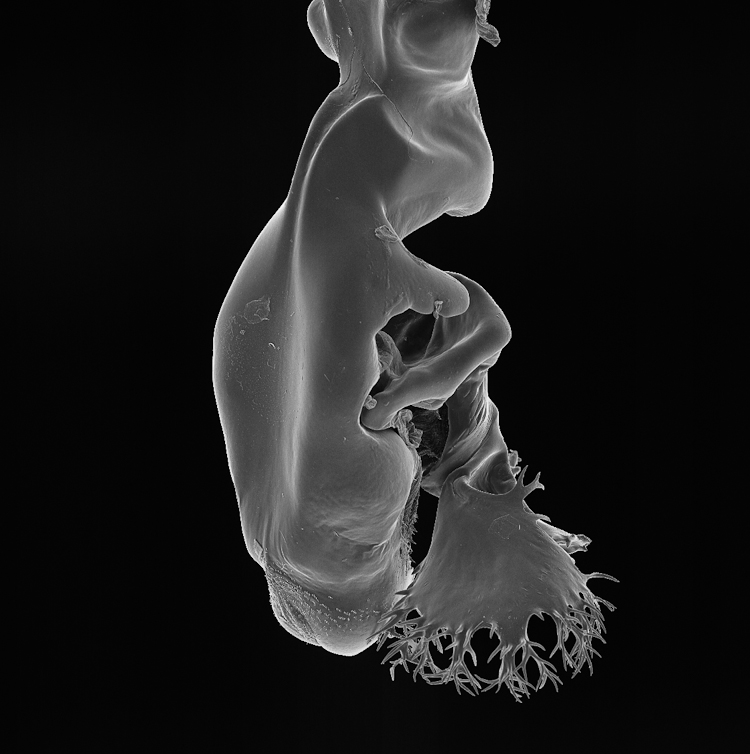

Supplement: Supplementary file 2 — rSEM illustrating the distiphallus of Oxysarcodexia (Xylocamptopsis) fringidea (Curran & Walley) (Sarcophagidae, Diptera); web-published using Magic 360TM script files. Click and drag to rotate the rSEM and point click to open and close the magnification tool. (doi: 10.3897/zookeys.328.5768.app2) File format: Hypertext Markup Document, archived (zip). [file ZooKeys-328-047-s002.zip › Fig S4 - magic360/fringidea/fringidea-sm-12.jpg]

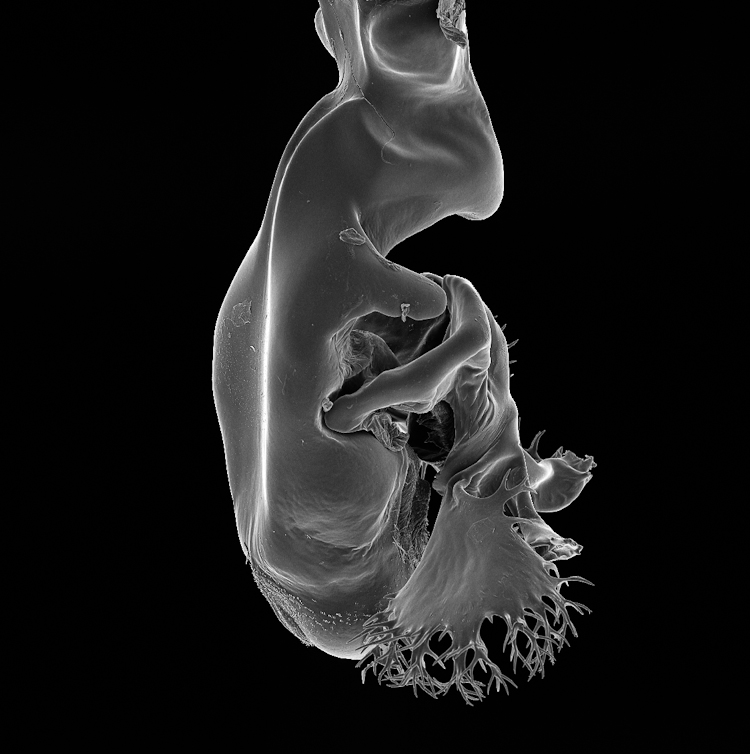

Supplement: Supplementary file 2 — rSEM illustrating the distiphallus of Oxysarcodexia (Xylocamptopsis) fringidea (Curran & Walley) (Sarcophagidae, Diptera); web-published using Magic 360TM script files. Click and drag to rotate the rSEM and point click to open and close the magnification tool. (doi: 10.3897/zookeys.328.5768.app2) File format: Hypertext Markup Document, archived (zip). [file ZooKeys-328-047-s002.zip › Fig S4 - magic360/fringidea/fringidea-sm-13.jpg]

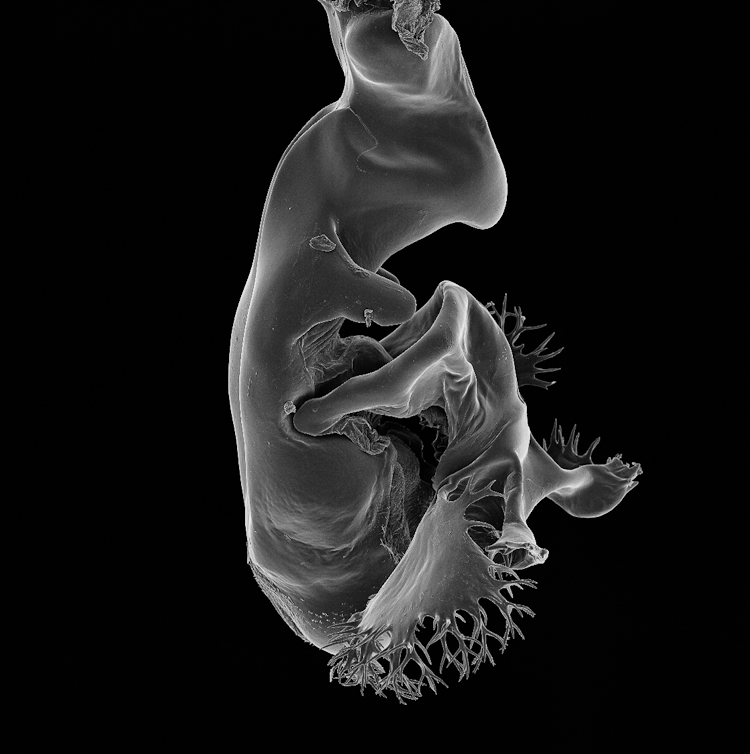

Supplement: Supplementary file 2 — rSEM illustrating the distiphallus of Oxysarcodexia (Xylocamptopsis) fringidea (Curran & Walley) (Sarcophagidae, Diptera); web-published using Magic 360TM script files. Click and drag to rotate the rSEM and point click to open and close the magnification tool. (doi: 10.3897/zookeys.328.5768.app2) File format: Hypertext Markup Document, archived (zip). [file ZooKeys-328-047-s002.zip › Fig S4 - magic360/fringidea/fringidea-sm-14.jpg]

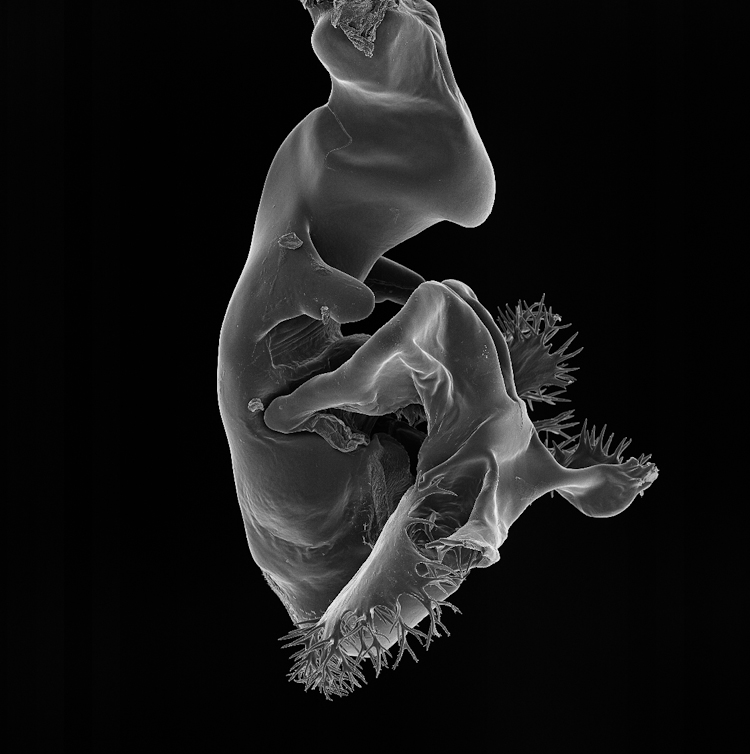

Supplement: Supplementary file 2 — rSEM illustrating the distiphallus of Oxysarcodexia (Xylocamptopsis) fringidea (Curran & Walley) (Sarcophagidae, Diptera); web-published using Magic 360TM script files. Click and drag to rotate the rSEM and point click to open and close the magnification tool. (doi: 10.3897/zookeys.328.5768.app2) File format: Hypertext Markup Document, archived (zip). [file ZooKeys-328-047-s002.zip › Fig S4 - magic360/fringidea/fringidea-sm-15.jpg]

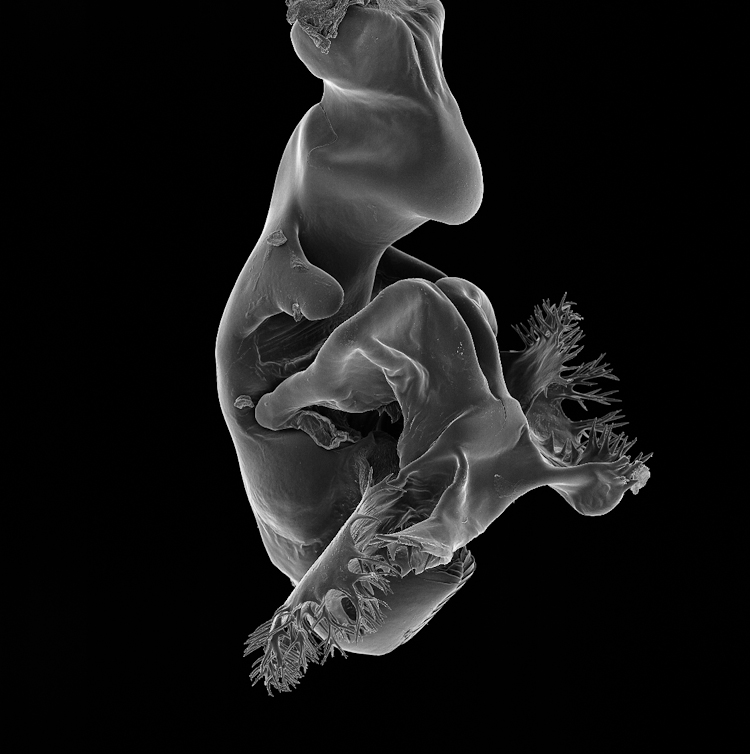

Supplement: Supplementary file 2 — rSEM illustrating the distiphallus of Oxysarcodexia (Xylocamptopsis) fringidea (Curran & Walley) (Sarcophagidae, Diptera); web-published using Magic 360TM script files. Click and drag to rotate the rSEM and point click to open and close the magnification tool. (doi: 10.3897/zookeys.328.5768.app2) File format: Hypertext Markup Document, archived (zip). [file ZooKeys-328-047-s002.zip › Fig S4 - magic360/fringidea/fringidea-sm-16.jpg]

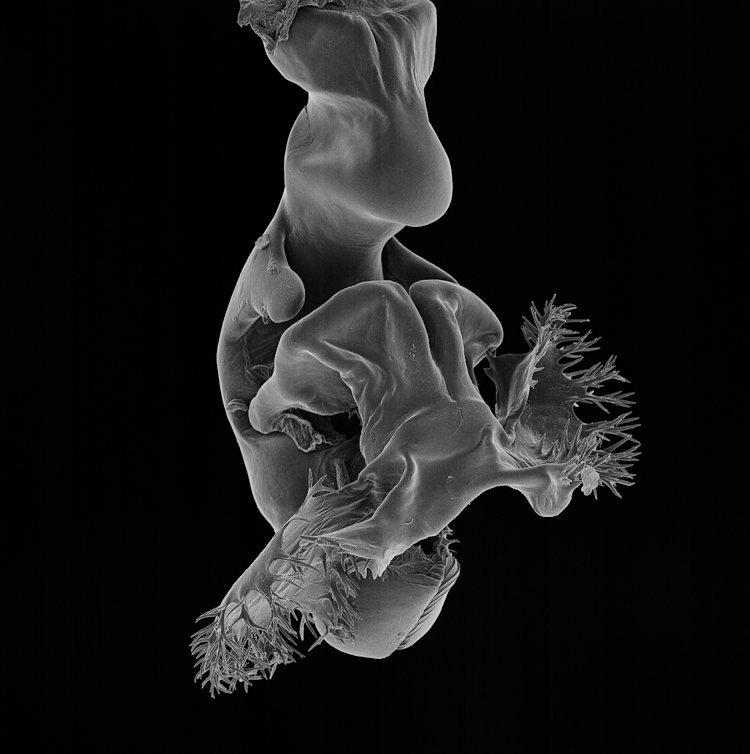

Supplement: Supplementary file 2 — rSEM illustrating the distiphallus of Oxysarcodexia (Xylocamptopsis) fringidea (Curran & Walley) (Sarcophagidae, Diptera); web-published using Magic 360TM script files. Click and drag to rotate the rSEM and point click to open and close the magnification tool. (doi: 10.3897/zookeys.328.5768.app2) File format: Hypertext Markup Document, archived (zip). [file ZooKeys-328-047-s002.zip › Fig S4 - magic360/fringidea/fringidea-sm-17.jpg]

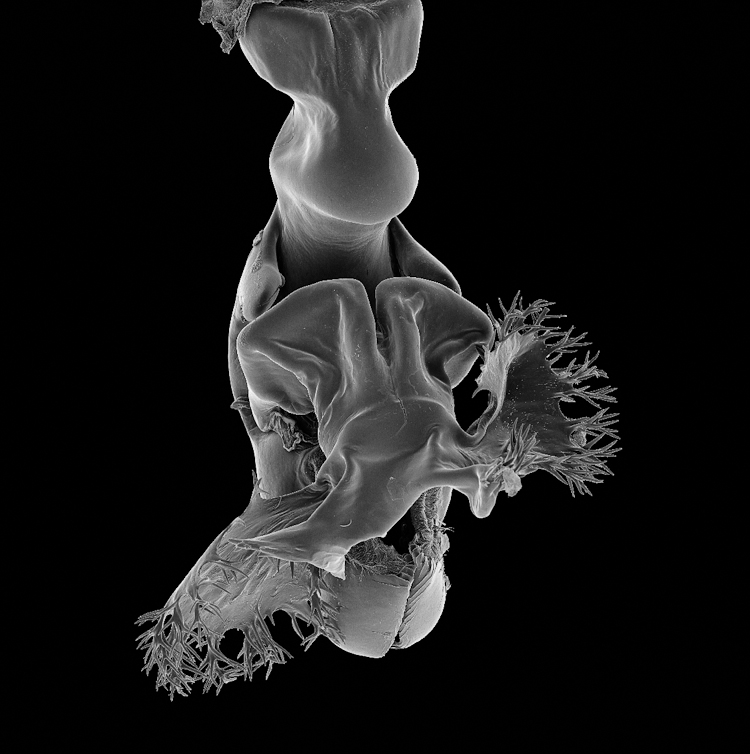

Supplement: Supplementary file 2 — rSEM illustrating the distiphallus of Oxysarcodexia (Xylocamptopsis) fringidea (Curran & Walley) (Sarcophagidae, Diptera); web-published using Magic 360TM script files. Click and drag to rotate the rSEM and point click to open and close the magnification tool. (doi: 10.3897/zookeys.328.5768.app2) File format: Hypertext Markup Document, archived (zip). [file ZooKeys-328-047-s002.zip › Fig S4 - magic360/fringidea/fringidea-sm-18.jpg]

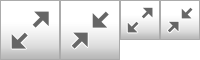

Supplement: Supplementary file 2 — rSEM illustrating the distiphallus of Oxysarcodexia (Xylocamptopsis) fringidea (Curran & Walley) (Sarcophagidae, Diptera); web-published using Magic 360TM script files. Click and drag to rotate the rSEM and point click to open and close the magnification tool. (doi: 10.3897/zookeys.328.5768.app2) File format: Hypertext Markup Document, archived (zip). [file ZooKeys-328-047-s002.zip › Fig S4 - magic360/magic360/graphics/buttons01.png]

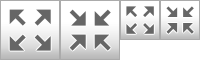

Supplement: Supplementary file 2 — rSEM illustrating the distiphallus of Oxysarcodexia (Xylocamptopsis) fringidea (Curran & Walley) (Sarcophagidae, Diptera); web-published using Magic 360TM script files. Click and drag to rotate the rSEM and point click to open and close the magnification tool. (doi: 10.3897/zookeys.328.5768.app2) File format: Hypertext Markup Document, archived (zip). [file ZooKeys-328-047-s002.zip › Fig S4 - magic360/magic360/graphics/buttons02.png]

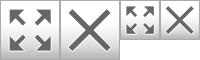

Supplement: Supplementary file 2 — rSEM illustrating the distiphallus of Oxysarcodexia (Xylocamptopsis) fringidea (Curran & Walley) (Sarcophagidae, Diptera); web-published using Magic 360TM script files. Click and drag to rotate the rSEM and point click to open and close the magnification tool. (doi: 10.3897/zookeys.328.5768.app2) File format: Hypertext Markup Document, archived (zip). [file ZooKeys-328-047-s002.zip › Fig S4 - magic360/magic360/graphics/buttons03.png]

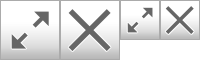

Supplement: Supplementary file 2 — rSEM illustrating the distiphallus of Oxysarcodexia (Xylocamptopsis) fringidea (Curran & Walley) (Sarcophagidae, Diptera); web-published using Magic 360TM script files. Click and drag to rotate the rSEM and point click to open and close the magnification tool. (doi: 10.3897/zookeys.328.5768.app2) File format: Hypertext Markup Document, archived (zip). [file ZooKeys-328-047-s002.zip › Fig S4 - magic360/magic360/graphics/buttons04.png]

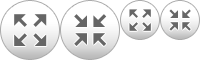

Supplement: Supplementary file 2 — rSEM illustrating the distiphallus of Oxysarcodexia (Xylocamptopsis) fringidea (Curran & Walley) (Sarcophagidae, Diptera); web-published using Magic 360TM script files. Click and drag to rotate the rSEM and point click to open and close the magnification tool. (doi: 10.3897/zookeys.328.5768.app2) File format: Hypertext Markup Document, archived (zip). [file ZooKeys-328-047-s002.zip › Fig S4 - magic360/magic360/graphics/buttons05.png]

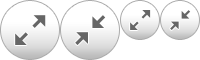

Supplement: Supplementary file 2 — rSEM illustrating the distiphallus of Oxysarcodexia (Xylocamptopsis) fringidea (Curran & Walley) (Sarcophagidae, Diptera); web-published using Magic 360TM script files. Click and drag to rotate the rSEM and point click to open and close the magnification tool. (doi: 10.3897/zookeys.328.5768.app2) File format: Hypertext Markup Document, archived (zip). [file ZooKeys-328-047-s002.zip › Fig S4 - magic360/magic360/graphics/buttons06.png]

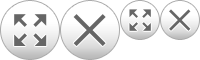

Supplement: Supplementary file 2 — rSEM illustrating the distiphallus of Oxysarcodexia (Xylocamptopsis) fringidea (Curran & Walley) (Sarcophagidae, Diptera); web-published using Magic 360TM script files. Click and drag to rotate the rSEM and point click to open and close the magnification tool. (doi: 10.3897/zookeys.328.5768.app2) File format: Hypertext Markup Document, archived (zip). [file ZooKeys-328-047-s002.zip › Fig S4 - magic360/magic360/graphics/buttons07.png]

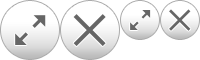

Supplement: Supplementary file 2 — rSEM illustrating the distiphallus of Oxysarcodexia (Xylocamptopsis) fringidea (Curran & Walley) (Sarcophagidae, Diptera); web-published using Magic 360TM script files. Click and drag to rotate the rSEM and point click to open and close the magnification tool. (doi: 10.3897/zookeys.328.5768.app2) File format: Hypertext Markup Document, archived (zip). [file ZooKeys-328-047-s002.zip › Fig S4 - magic360/magic360/graphics/buttons08.png]

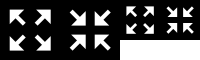

Supplement: Supplementary file 2 — rSEM illustrating the distiphallus of Oxysarcodexia (Xylocamptopsis) fringidea (Curran & Walley) (Sarcophagidae, Diptera); web-published using Magic 360TM script files. Click and drag to rotate the rSEM and point click to open and close the magnification tool. (doi: 10.3897/zookeys.328.5768.app2) File format: Hypertext Markup Document, archived (zip). [file ZooKeys-328-047-s002.zip › Fig S4 - magic360/magic360/graphics/buttons09.png]

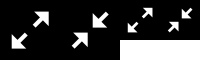

Supplement: Supplementary file 2 — rSEM illustrating the distiphallus of Oxysarcodexia (Xylocamptopsis) fringidea (Curran & Walley) (Sarcophagidae, Diptera); web-published using Magic 360TM script files. Click and drag to rotate the rSEM and point click to open and close the magnification tool. (doi: 10.3897/zookeys.328.5768.app2) File format: Hypertext Markup Document, archived (zip). [file ZooKeys-328-047-s002.zip › Fig S4 - magic360/magic360/graphics/buttons10.png]

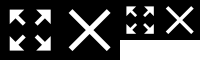

Supplement: Supplementary file 2 — rSEM illustrating the distiphallus of Oxysarcodexia (Xylocamptopsis) fringidea (Curran & Walley) (Sarcophagidae, Diptera); web-published using Magic 360TM script files. Click and drag to rotate the rSEM and point click to open and close the magnification tool. (doi: 10.3897/zookeys.328.5768.app2) File format: Hypertext Markup Document, archived (zip). [file ZooKeys-328-047-s002.zip › Fig S4 - magic360/magic360/graphics/buttons11.png]

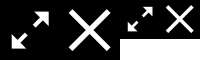

Supplement: Supplementary file 2 — rSEM illustrating the distiphallus of Oxysarcodexia (Xylocamptopsis) fringidea (Curran & Walley) (Sarcophagidae, Diptera); web-published using Magic 360TM script files. Click and drag to rotate the rSEM and point click to open and close the magnification tool. (doi: 10.3897/zookeys.328.5768.app2) File format: Hypertext Markup Document, archived (zip). [file ZooKeys-328-047-s002.zip › Fig S4 - magic360/magic360/graphics/buttons12.png]

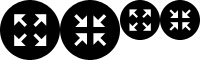

Supplement: Supplementary file 2 — rSEM illustrating the distiphallus of Oxysarcodexia (Xylocamptopsis) fringidea (Curran & Walley) (Sarcophagidae, Diptera); web-published using Magic 360TM script files. Click and drag to rotate the rSEM and point click to open and close the magnification tool. (doi: 10.3897/zookeys.328.5768.app2) File format: Hypertext Markup Document, archived (zip). [file ZooKeys-328-047-s002.zip › Fig S4 - magic360/magic360/graphics/buttons13.png]

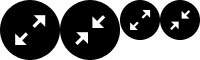

Supplement: Supplementary file 2 — rSEM illustrating the distiphallus of Oxysarcodexia (Xylocamptopsis) fringidea (Curran & Walley) (Sarcophagidae, Diptera); web-published using Magic 360TM script files. Click and drag to rotate the rSEM and point click to open and close the magnification tool. (doi: 10.3897/zookeys.328.5768.app2) File format: Hypertext Markup Document, archived (zip). [file ZooKeys-328-047-s002.zip › Fig S4 - magic360/magic360/graphics/buttons14.png]

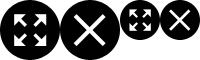

Supplement: Supplementary file 2 — rSEM illustrating the distiphallus of Oxysarcodexia (Xylocamptopsis) fringidea (Curran & Walley) (Sarcophagidae, Diptera); web-published using Magic 360TM script files. Click and drag to rotate the rSEM and point click to open and close the magnification tool. (doi: 10.3897/zookeys.328.5768.app2) File format: Hypertext Markup Document, archived (zip). [file ZooKeys-328-047-s002.zip › Fig S4 - magic360/magic360/graphics/buttons15.png]

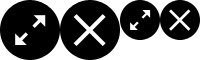

Supplement: Supplementary file 2 — rSEM illustrating the distiphallus of Oxysarcodexia (Xylocamptopsis) fringidea (Curran & Walley) (Sarcophagidae, Diptera); web-published using Magic 360TM script files. Click and drag to rotate the rSEM and point click to open and close the magnification tool. (doi: 10.3897/zookeys.328.5768.app2) File format: Hypertext Markup Document, archived (zip). [file ZooKeys-328-047-s002.zip › Fig S4 - magic360/magic360/graphics/buttons16.png]

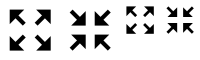

Supplement: Supplementary file 2 — rSEM illustrating the distiphallus of Oxysarcodexia (Xylocamptopsis) fringidea (Curran & Walley) (Sarcophagidae, Diptera); web-published using Magic 360TM script files. Click and drag to rotate the rSEM and point click to open and close the magnification tool. (doi: 10.3897/zookeys.328.5768.app2) File format: Hypertext Markup Document, archived (zip). [file ZooKeys-328-047-s002.zip › Fig S4 - magic360/magic360/graphics/buttons17.png]

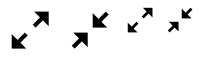

Supplement: Supplementary file 2 — rSEM illustrating the distiphallus of Oxysarcodexia (Xylocamptopsis) fringidea (Curran & Walley) (Sarcophagidae, Diptera); web-published using Magic 360TM script files. Click and drag to rotate the rSEM and point click to open and close the magnification tool. (doi: 10.3897/zookeys.328.5768.app2) File format: Hypertext Markup Document, archived (zip). [file ZooKeys-328-047-s002.zip › Fig S4 - magic360/magic360/graphics/buttons18.png]

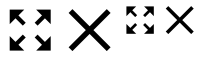

Supplement: Supplementary file 2 — rSEM illustrating the distiphallus of Oxysarcodexia (Xylocamptopsis) fringidea (Curran & Walley) (Sarcophagidae, Diptera); web-published using Magic 360TM script files. Click and drag to rotate the rSEM and point click to open and close the magnification tool. (doi: 10.3897/zookeys.328.5768.app2) File format: Hypertext Markup Document, archived (zip). [file ZooKeys-328-047-s002.zip › Fig S4 - magic360/magic360/graphics/buttons19.png]

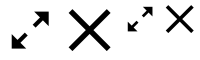

Supplement: Supplementary file 2 — rSEM illustrating the distiphallus of Oxysarcodexia (Xylocamptopsis) fringidea (Curran & Walley) (Sarcophagidae, Diptera); web-published using Magic 360TM script files. Click and drag to rotate the rSEM and point click to open and close the magnification tool. (doi: 10.3897/zookeys.328.5768.app2) File format: Hypertext Markup Document, archived (zip). [file ZooKeys-328-047-s002.zip › Fig S4 - magic360/magic360/graphics/buttons20.png]

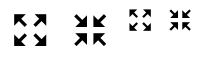

Supplement: Supplementary file 2 — rSEM illustrating the distiphallus of Oxysarcodexia (Xylocamptopsis) fringidea (Curran & Walley) (Sarcophagidae, Diptera); web-published using Magic 360TM script files. Click and drag to rotate the rSEM and point click to open and close the magnification tool. (doi: 10.3897/zookeys.328.5768.app2) File format: Hypertext Markup Document, archived (zip). [file ZooKeys-328-047-s002.zip › Fig S4 - magic360/magic360/graphics/buttons21.png]

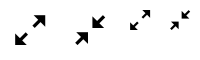

Supplement: Supplementary file 2 — rSEM illustrating the distiphallus of Oxysarcodexia (Xylocamptopsis) fringidea (Curran & Walley) (Sarcophagidae, Diptera); web-published using Magic 360TM script files. Click and drag to rotate the rSEM and point click to open and close the magnification tool. (doi: 10.3897/zookeys.328.5768.app2) File format: Hypertext Markup Document, archived (zip). [file ZooKeys-328-047-s002.zip › Fig S4 - magic360/magic360/graphics/buttons22.png]

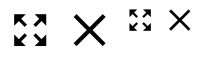

Supplement: Supplementary file 2 — rSEM illustrating the distiphallus of Oxysarcodexia (Xylocamptopsis) fringidea (Curran & Walley) (Sarcophagidae, Diptera); web-published using Magic 360TM script files. Click and drag to rotate the rSEM and point click to open and close the magnification tool. (doi: 10.3897/zookeys.328.5768.app2) File format: Hypertext Markup Document, archived (zip). [file ZooKeys-328-047-s002.zip › Fig S4 - magic360/magic360/graphics/buttons23.png]

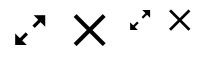

Supplement: Supplementary file 2 — rSEM illustrating the distiphallus of Oxysarcodexia (Xylocamptopsis) fringidea (Curran & Walley) (Sarcophagidae, Diptera); web-published using Magic 360TM script files. Click and drag to rotate the rSEM and point click to open and close the magnification tool. (doi: 10.3897/zookeys.328.5768.app2) File format: Hypertext Markup Document, archived (zip). [file ZooKeys-328-047-s002.zip › Fig S4 - magic360/magic360/graphics/buttons24.png]

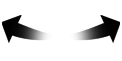

Supplement: Supplementary file 2 — rSEM illustrating the distiphallus of Oxysarcodexia (Xylocamptopsis) fringidea (Curran & Walley) (Sarcophagidae, Diptera); web-published using Magic 360TM script files. Click and drag to rotate the rSEM and point click to open and close the magnification tool. (doi: 10.3897/zookeys.328.5768.app2) File format: Hypertext Markup Document, archived (zip). [file ZooKeys-328-047-s002.zip › Fig S4 - magic360/magic360/graphics/hint-01.png]

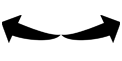

Supplement: Supplementary file 2 — rSEM illustrating the distiphallus of Oxysarcodexia (Xylocamptopsis) fringidea (Curran & Walley) (Sarcophagidae, Diptera); web-published using Magic 360TM script files. Click and drag to rotate the rSEM and point click to open and close the magnification tool. (doi: 10.3897/zookeys.328.5768.app2) File format: Hypertext Markup Document, archived (zip). [file ZooKeys-328-047-s002.zip › Fig S4 - magic360/magic360/graphics/hint-02.png]

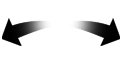

Supplement: Supplementary file 2 — rSEM illustrating the distiphallus of Oxysarcodexia (Xylocamptopsis) fringidea (Curran & Walley) (Sarcophagidae, Diptera); web-published using Magic 360TM script files. Click and drag to rotate the rSEM and point click to open and close the magnification tool. (doi: 10.3897/zookeys.328.5768.app2) File format: Hypertext Markup Document, archived (zip). [file ZooKeys-328-047-s002.zip › Fig S4 - magic360/magic360/graphics/hint-03.png]

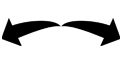

Supplement: Supplementary file 2 — rSEM illustrating the distiphallus of Oxysarcodexia (Xylocamptopsis) fringidea (Curran & Walley) (Sarcophagidae, Diptera); web-published using Magic 360TM script files. Click and drag to rotate the rSEM and point click to open and close the magnification tool. (doi: 10.3897/zookeys.328.5768.app2) File format: Hypertext Markup Document, archived (zip). [file ZooKeys-328-047-s002.zip › Fig S4 - magic360/magic360/graphics/hint-04.png]

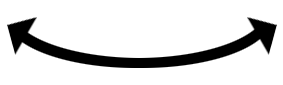

Supplement: Supplementary file 2 — rSEM illustrating the distiphallus of Oxysarcodexia (Xylocamptopsis) fringidea (Curran & Walley) (Sarcophagidae, Diptera); web-published using Magic 360TM script files. Click and drag to rotate the rSEM and point click to open and close the magnification tool. (doi: 10.3897/zookeys.328.5768.app2) File format: Hypertext Markup Document, archived (zip). [file ZooKeys-328-047-s002.zip › Fig S4 - magic360/magic360/graphics/hint-05.png]

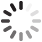

Supplement: Supplementary file 2 — rSEM illustrating the distiphallus of Oxysarcodexia (Xylocamptopsis) fringidea (Curran & Walley) (Sarcophagidae, Diptera); web-published using Magic 360TM script files. Click and drag to rotate the rSEM and point click to open and close the magnification tool. (doi: 10.3897/zookeys.328.5768.app2) File format: Hypertext Markup Document, archived (zip). [file ZooKeys-328-047-s002.zip › Fig S4 - magic360/magic360/graphics/loader.gif]

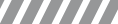

Supplement: Supplementary file 2 — rSEM illustrating the distiphallus of Oxysarcodexia (Xylocamptopsis) fringidea (Curran & Walley) (Sarcophagidae, Diptera); web-published using Magic 360TM script files. Click and drag to rotate the rSEM and point click to open and close the magnification tool. (doi: 10.3897/zookeys.328.5768.app2) File format: Hypertext Markup Document, archived (zip). [file ZooKeys-328-047-s002.zip › Fig S4 - magic360/magic360/graphics/progress.gif]
